# Antimicrobial resistance among Gram-negative agents of bacteraemia in the UK and Ireland: trends from 2001 to 2019

**DOI:** 10.1093/jac/dkaf250

**Published:** 2025-10-27

**Authors:** Rosy Reynolds, Shazad Mushtaq, Russell Hope, Carolyne Horner, Rachael Adkin, Aiysha Chaudhry, Olisaeloka Nsonwu, Michael Allen, Christopher Longshaw, Benjamin J Parcell, David M Livermore

**Affiliations:** Population Health Sciences, University of Bristol, Bristol BS8 2PS, UK; British Society for Antimicrobial Chemotherapy, 53 Regent Place, Birmingham B1 3NJ, UK; Antimicrobial Resistance and Healthcare Associated Infections Reference Unit, UK Health Security Agency, Colindale, London NW9 5EQ, UK; Antimicrobial Resistance and Healthcare Associated Infections Division, UK Health Security Agency, Colindale, London NW9 5EQ, UK; British Society for Antimicrobial Chemotherapy, 53 Regent Place, Birmingham B1 3NJ, UK; Antimicrobial Resistance and Healthcare Associated Infections Reference Unit, UK Health Security Agency, Colindale, London NW9 5EQ, UK; Antimicrobial Resistance and Healthcare Associated Infections Reference Unit, UK Health Security Agency, Colindale, London NW9 5EQ, UK; Antimicrobial Resistance and Healthcare Associated Infections Division, UK Health Security Agency, Colindale, London NW9 5EQ, UK; British Society for Antimicrobial Chemotherapy, 53 Regent Place, Birmingham B1 3NJ, UK; Medical Affairs, MSD (UK) Limited, 120 Moorgate, London EC2M 6UR, UK; British Society for Antimicrobial Chemotherapy, 53 Regent Place, Birmingham B1 3NJ, UK; Scientific Affairs, Shionogi B.V., Fifty Paddington, 50 Eastbourne Terrace, Paddington W2 6LG, UK; Division of Population Health and Genomics, School of Medicine, University of Dundee, Ninewells Hospital and Medical School, Dundee DD1 9SY, UK; Department of Medical Microbiology, Ninewells Hospital and Medical School, Dundee DD1 9SY, UK; Antimicrobial Resistance and Healthcare Associated Infections Reference Unit, UK Health Security Agency, Colindale, London NW9 5EQ, UK; Norwich Medical School, University of East Anglia, Norwich NR4 7TJ, UK

## Abstract

**Objectives:**

The BSAC Bacteraemia Resistance Surveillance Programme collected isolates from UK and Irish hospitals for central testing. Concurrent UKHSA surveillance collected English hospitals’ own susceptibility data. Results were reviewed and compared.

**Methods:**

The BSAC surveillance collected fixed quotas of isolates per site annually from 2001 to 2019. MIC testing was by BSAC agar dilution. Resistance mechanisms were investigated by synergy tests, interpretive reading and PCR. The UKHSA seeks data on all bacteraemia isolates in England.

**Results:**

For *Escherichia coli*, which now causes >30% of all bacteraemias, there were marked early (2002–06) rises in resistance to cephalosporins, fluoroquinolones and gentamicin, followed by small falls, stabilization, then from around 2015, very slow rises, with similar patterns seen for *Klebsiella pneumoniae*. Most cephalosporin resistance in these two species involved ESBLs, principally CTX-M types. Both species had frequent co-amoxiclav resistance. Cephalosporin resistance—mostly AmpC-mediated—declined in *Enterobacter* and *Serratia* spp., as did fluoroquinolone resistance, likely reflecting reduced use and selection pressure. Proteeae showed few changes; increasing dominance of *Proteus mirabilis* in the BSAC collection was not confirmed by the UKHSA dataset. Resistance in *Pseudomonas aeruginosa* was uncommon and showed little temporal change in either dataset. Carbapenemases remained extremely rare in all species. Newer and developmental agents covered many resistance types, but none covered all types.

**Conclusions:**

Except for early rises of cephalosporin, fluoroquinolone and gentamicin resistance in *E. coli* and *K. pneumoniae*, there was little evidence for rising resistance and some evidence of declining resistance, notably in species where it predominantly involves AmpC derepression.

## Introduction

Before the antibiotic era, Gram-negative bacteria caused only 10% of bacteraemias. Mortality was lower than among the many more bacteraemias caused by Gram-positives, principally *Streptococcus pyogenes*.^1^ Modern medicine changed this aetiology. ‘Typical’ bacteraemia patients are now older, have more underlying disease and—with antibiotics—are better protected against Gram-positive pathogens. On the other hand, owing to immune compromise, many more patients are vulnerable to Gram-negative opportunists, which now cause *c.* 50% of all bacteraemias.^2^  *Escherichia coli* alone accounts for one-third, up from one-fifth in Year 2000.^3^ Other prominent agents include *Klebsiella*, *Enterobacter*, *Pseudomonas* and *Serratia* spp., also Proteeae.^2^ Many *E. coli* and Proteeae bacteraemias have a community onset; other Gram-negative opportunists are largely nosocomial.^4^

Gram-negative bacteria are inherently less susceptible to antibiotics than Gram-positives, reflecting their outer wall structure, which acts cooperatively with efflux and antibiotic-degrading enzymes to prevent cellular antibiotic levels from rising.^5^ Mutations can up-regulate efflux and/or cause porin loss, further increasing resistance; other mutations up-regulate antibiotic-degrading enzymes.^6^ Even more importantly, Gram-negative bacteria, particularly Enterobacterales, now host numerous plasmids encoding further resistances. Major international shifts occurred during the two decades of BSAC bacteraemia surveillance (2001–19), notably: (i) a rise of fluoroquinolone resistance among Gram-negative bacteria,^7,8^ (ii) spread of CTX-M ESBLs and multiresistant *E. coli* ST131^9,10^ and (iii) emergence of carbapenemases.^11^

Development of new therapies has been slower than against Gram-positive pathogens, reflecting the challenge of finding compounds that efficiently permeate Gram-negative bacteria. Among agents launched in 2001–10, the first decade of the BSAC’s bacteraemia surveillance, only tigecycline and doripenem had anti-Gram-negative activity. Since 2015, these have been joined by ceftolozane/tazobactam, ceftazidime/avibactam, imipenem/relebactam, meropenem/vaborbactam, cefiderocol, plazomicin and eravacycline, all with anti-Gram-negative activity, covering some or many critical resistance types. This paper charts resistance trends among Gram-negative pathogens in the UK and Ireland from 2001 to 2019, using both BSAC and UKHSA data, and reviews the activity of those newer agents included in the Programme.

## Materials and methods

Methods for the BSAC and UKHSA surveillances are fully described elsewhere in this supplement^12^; skeleton details are provided below.

The BSAC surveillance sought up to 250 isolates per species group annually from 25 laboratories, increasing to 280 from 40 laboratories in 2010–15. For *E. coli*, these totals were doubled from 2008 onwards (i.e. to 500 in 2008–09 and 2016–19 and 560 in 2010–15). *Serratia* were collected in a mixed ‘other Gram-negatives’ group until 2007 but as a specified group thereafter. Isolates of *Klebsiella aerogenes* were known and collected as *Enterobacter aerogenes* until 2018, counting in the *Enterobacter* quotas, and then reassigned to *Klebsiella*. Tables S1–S3 (available as Supplementary data at *JAC* Online) detail numbers of laboratories contributing isolates annually and necessary data exclusions.

Bacterial identification was originally by classical methods, but later shifted to MALDI-TOF. MICs were determined by BSAC agar dilution and categorized against EUCAST 2022 breakpoints. β-Lactamase genes were sought by PCR; resistance mechanisms were inferred based upon these results and interpretive reading of MIC data. The antibiotics tested included core agents tested in all years under the aegis of the BSAC, as well as those included for variable periods contingent on sponsorship by funders. Tables S4–S10 detail breakpoints (EUCAST v12.0, 2022) and susceptibility tests by organism, antimicrobial and years included. Not all antimicrobials were tested every year. Tables S11–S14 and Figures S1–S3 cover patient characteristics, noting any missing data. MIC distributions are also presented.

### Analysis

Analysis was descriptive and largely graphical, using Stata 18.0 (StataCorp LLC: College Station, TX, USA) and Bischoff’s colour vision-sensitive ‘plotplainblind’ graph scheme.^13^ Missing data were excluded in the calculation of percentages.

## Results

### BSAC isolate collection

The collection included 7796 isolates of *E. coli* and from 2346 to 5108 isolates of each of five other groups (Table S2). The proportion of male patients varied from 48% for *E. coli* to 64% for *Serratia* spp (Table S11). The median age was lowest for *Enterobacter* (63 years) and highest for Proteeae (76 years) (Table S12 and Figure S1); proportions of patients aged ≥80 years ranged from 20% (*Serratia*) to 38% (Proteeae) (Table S12). All organism groups included a distinct subgroup of isolates from infants <1 year old, largest for *Enterobacter* (6%) (Table S12). The proportion of ‘hospital-onset’ isolates, from patients hospitalized for >48 h, declined over time, particularly after 2008, but was consistently lowest for *E. coli* and Proteeae—which also had fewest ICU samples—and highest for *Enterobacter* and *Serratia* (Table S13 and Figures S2 and S3). Data for sources of infection were limited to 2001–13 and were incompletely reported; the most frequently recorded were invasive devices for *Serratia* and *Enterobacter* and the genitourinary tract for other Enterobacterales and *Pseudomonas*, without clear time trends (Table S14).

### UKHSA data extract

Numbers of bacteraemia reports for each organism group increased over time, with antibiotic susceptibility data increasingly included (Table S15 and Figures S4 and S5). During the period of comparison with BSAC data (i.e. 2001–19), annual numbers of reports ranged from 11 423 to 39 907 for *E. coli*, and—among other groups—from 556 (*Serratia*, 2001) to 10 391 (*Klebsiella*, 2019). Reports for *E. coli* and *Klebsiella* increased greatly between 2001 and 2019 (3.5- and 3.2-fold, respectively), those for other organism groups rose between 1.5-fold (*Enterobacter*) and 2.4-fold (Proteeae).

### E. coli

**Figure 1.**
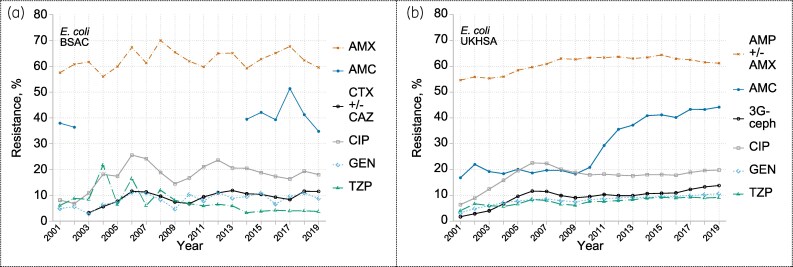
Resistance trends among *E. coli* from bacteraemia in (a) BSAC and (b) UKHSA surveillance. Resistance to: AMC co-amoxiclav; AMP +/- AMX one or both of ampicillin and amoxicillin; AMX amoxicillin; CTX +/- CAZ one or both of cefotaxime and ceftazidime; CIP ciprofloxacin; GEN gentamicin; TZP piperacillin/tazobactam; 3G-ceph any one or more of CTX, CAZ or (in UKHSA data only and very rarely tested) cefotetan. The break in BSAC co-amoxiclav data reflects the period when only the 2:1 amoxicillin:clavulanate combination was tested: the resulting data cannot be analysed against the current breakpoint, which is based on testing with fixed 2 mg/L of clavulanate.

The large number of isolates in the UKHSA dataset (see Tables S2 and S15) enabled robust description of resistance trends, supported and extended by BSAC data (Figure 1). The UKHSA data show resistance increasing sharply from 2001 to a peak in 2006–07 for third-generation cephalosporins (1.7% rising to 11.6%), ciprofloxacin (6.4% to 22.5%) and gentamicin (3.2% to 8.5%). After minor subsequent falls from 2006–07 to 2009–10 and a period of stability at *c.* 10% for cephalosporins, 18% for ciprofloxacin and 8% for gentamicin, resistance rose marginally, reaching 13.8% for cephalosporins, 19.8% for ciprofloxacin and 10.6% for gentamicin by 2019. Aminopenicillin resistance rates always exceeded 50% and were consistently >60% from 2007; those for piperacillin/tazobactam stabilized at around 9% in 2014–19 in UKHSA data but were lower (4%) in BSAC data. A sudden increase in co-amoxiclav resistance from *c.* 20% in 2001–10 to ≥40% in 2014–19 in UKHSA data likely reflects changing breakpoints and testing modalities; no such shift was apparent in BSAC data when comparing the two periods (2001–02 and 2014 onwards) when co-amoxiclav was tested with a fixed concentration of 2 mg/L clavulanate.^14^

**Figure 2.**
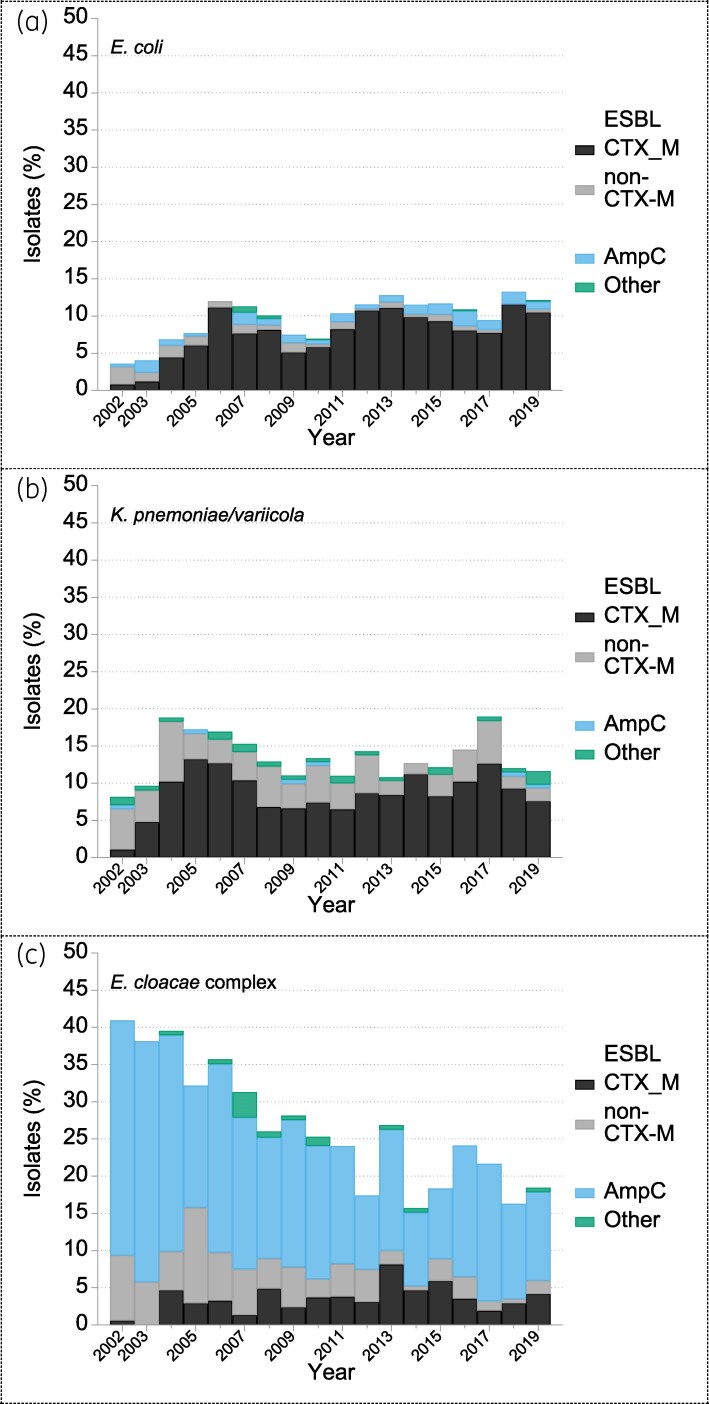
Mechanisms of resistance to third-generation cephalosporins in isolates of (a) *E. coli*, (b) *K. pneumoniae*/*variicola* and (c) *E. cloacae* complex from BSAC bacteraemia surveillance over time. Resistance to third-generation cephalosporins was recorded if the cefotaxime MIC  was > 2 mg/L, the ceftazidime MIC  > 4 mg/L or if ESBL or AmpC production was inferred from synergy tests and interpretive reading. Isolates were allocated to mechanism categories in order: any with CTX-M ESBL, then ESBL (but not a CTX-M type), AmpC (but no ESBL) and ‘other’ mechanisms (not ESBL or AmpC).

ESBL production, detected by cephalosporin/clavulanate synergy and PCR for *bla*_CTX-M_, tracked cefotaxime resistance, increasing from 3% in 2001–04 to 10% in 2015–19 (Figure 2). The proportion of ESBL producers with CTX-M enzymes rose from 59% in 2002–04 to around 95% from 2010 onwards. Isolates with copious AmpC (identified from 2002–19, primarily by cefotaxime/cloxacillin synergy) remained rare, averaging 1% of all *E. coli*.

Ceftobiprole resistance, monitored from 2004 to 2019, tracked cefotaxime. Among aminoglycosides, tobramycin resistance rates (monitored 2014–19) tracked gentamicin. Resistance to trimethoprim, tested from 2014 to 2019, averaged 38% (detail not shown).

Nine antimicrobials (not all tested every year) had <1% resistance prevalence and mode MICs as follows: ceftazidime/avibactam (0.12 mg/L), ceftolozane/tazobactam (0.12 mg/L), ertapenem (0.008 mg/L), imipenem (0.12 mg/L), imipenem/relebactam (0.12 mg/L), meropenem (0.015 mg/L), amikacin (1 mg/L), colistin (0.5 mg/L) and tigecycline (0.25 mg/L) (see MIC distributions in the Appendix to the Supplementary Data).

Only two *E. coli* were confirmed to produce carbapenemases—one with an OXA-48-like enzyme in 2015 and one with an NDM type in 2019; two isolates from 2006 had phenotypes suggesting carbapenemases, but no relevant genes were found despite extensive PCR testing.

### Klebsiella *spp.*

#### K. pneumoniae

*Klebsiella pneumoniae* (including a few *Klebsiella variicola* isolates as identified by MALDI-TOF) outnumbered *Klebsiella oxytoca* by *c.* 3.5:1 in the BSAC surveillance, with little change over time (see Tables S4 and S6); the ratio for the recent-most five years of UKHSA data was comparable, at *c.* 4.5:1. The proportion of *K. aerogenes* (formerly *E. aerogenes*) was small at around 4%, based upon BSAC surveillance in 2019 and UKHSA data for 2015–19; this proportion could not be estimated for earlier years because the BSAC surveillance collected *K. aerogenes* in the *Enterobacter* quota until 2018. In early years of the UKHSA surveillance, some *K. pneumoniae* were recorded as ‘*K. aerogenes*’, following an older informal taxonomy, precluding confident calculation of species proportions.

**Figure 3.**
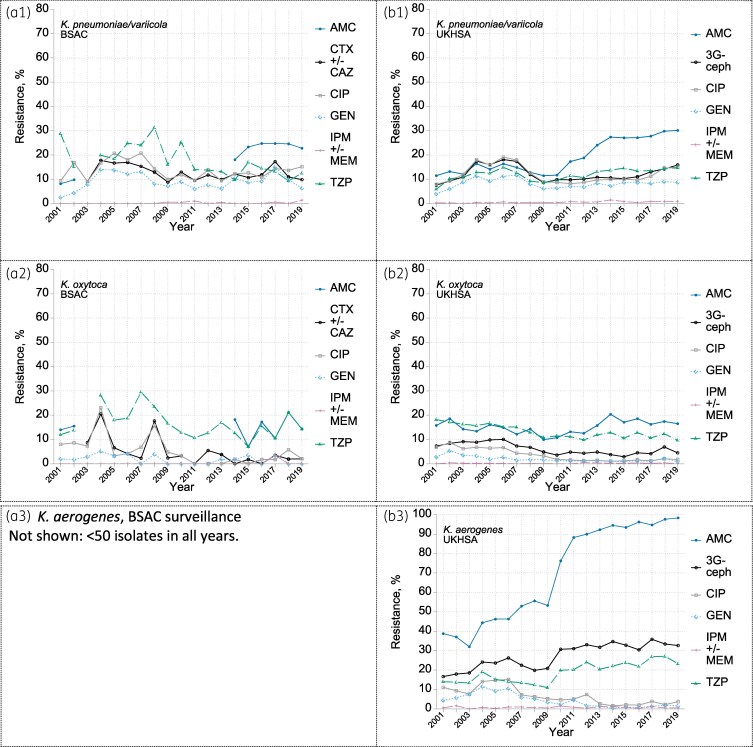
Resistance trends among (a) *K. pneumoniae*/*variicola*, (b) *K. oxytoca* and (c) *K. aerogenes* from bacteraemia in (1) BSAC and (2) UKHSA surveillances. Resistance to: AMC co-amoxiclav; CTX +/- CAZ one or both of cefotaxime and ceftazidime; CIP ciprofloxacin; GEN gentamicin; IPM +/- MEM one or both of imipenem and meropenem; TZP piperacillin/tazobactam; 3G-ceph any one or more of CTX, CAZ and (in UKHSA data only and very rarely tested) cefotetan. Note the very small *N* of isolates for BSAC *K. oxytoca* (range 36–69/year); BSAC *K. aerogenes* not shown (*N* < 50 in all years). For UKHSA ‘*K. aerogenes*’, it should be assumed that isolates in the early surveillance years should more correctly have been called *K. pneumoniae*, whereas those more recently identified as ‘*K. aerogenes*’ would previously have been identified as ‘*E. aerogenes*’. The large step in co-amoxiclav resistance around 2009–11 indicates the date of these shifts (see text). Breaks in the BSAC co-amoxiclav data reflect the period when only the 2:1 amoxicillin:clavulanate combination was tested: the resulting data cannot be analysed against the current breakpoint, which is based on testing with a fixed 2 mg/L of clavulanate.

For *K. pneumoniae/variicola*, the UKHSA dataset showed clear early rises in the prevalence of resistance to third-generation cephalosporins, ciprofloxacin, gentamicin and piperacillin/tazobactam from 4%–8% in 2001 to 10%–20% in 2004–07. These rates then drifted downwards towards 2010 (Figure 3); thereafter, gentamicin resistance prevalence remained just below 10%, whereas the cephalosporin, ciprofloxacin and piperacillin/tazobactam resistance rates rebounded, reaching *c.* 15% by 2019. Co-amoxiclav resistance, relatively stable in the early 2000s, almost tripled in the UKHSA extract after 2010 before levelling at 30% in 2018–19. As with *E. coli*, this shift likely reflects changing testing modalities. Nonetheless, and unlike for *E. coli*, the smaller BSAC dataset also indicates a real rise in resistance, as tested with a fixed 2 mg/L clavulanate, between the 2001–02 and 2014–19 periods.

Resistance remained <1.5% for ertapenem and <1% for other carbapenems and carbapenem/inhibitor combinations in both the BSAC and UKHSA datasets (Figure 3 and MIC plots in the Supplementary data). Resistance also was very rare for amikacin (<1%; mode MIC, 1 mg/L), colistin (1.3%; 0.5 mg/L), ceftazidime/avibactam (<1%; 0.25 mg/L) and ceftolozane/tazobactam (<1%; 0.25 mg/L); the prevalence of resistance to tobramycin (tested 2014–19) mirrored that for gentamicin. The mode MIC for tigecycline, which has no EUCAST breakpoint for the genus, was 0.5 mg/L (see MIC distributions in the Appendix to the Supplementary Data).

Proportions of *K. pneumoniae* resistant to ceftazidime and cefotaxime tracked the proportion of ESBL producers, but resistance was more frequent to ceftobiprole, ceftaroline and cefuroxime (detail not shown), all with breakpoints closer to the modal MICs for susceptible isolates than, for example, cefotaxime. AmpC was rarely seen (0.2% overall; Figure 2). Among ESBL producers, the proportion with CTX-M type enzymes rose rapidly from 17% (2/12) in 2002 to average of 73% from 2005 to 2019 (Figure 2). Carbapenemases were sought formally from 2009 and detected in 18 isolates up to 2019; seven had OXA-48-like enzymes, five had NDM, four had VIM, and two had KPC types. There was no evident time trend, except maybe that one-third (6/18; three each with OXA-48-like and NDM enzymes) were from the final surveillance year (2019).

#### K. oxytoca

UKHSA surveillance showed different resistance trends in *K. oxytoca* versus *K. pneumoniae*. Rather than rising, already sub-10% rates of resistance fell, stabilizing in 2010–19 at <2% for ciprofloxacin and gentamicin and *c.* 5% for third-generation cephalosporins. Piperacillin/tazobactam resistance receded from 18% in 2001 to 11% by 2019 (Figure 3). By contrast, after falling to a brief low around 10% in 2009–10, resistance to co-amoxiclav rose to a steady 16%–18% in 2015–19, doubtless again reflecting changed testing. Minimal imipenem or meropenem resistance (<1%) was seen throughout.

Annual resistance prevalence estimates were very variable in the BSAC dataset, reflecting the few *K. oxytoca* collected. Nonetheless, the data are useful in aggregate for antimicrobials seldom reported in UKHSA data. As for *K. pneumoniae*, resistance was similarly prevalent for tobramycin and gentamicin (*c*. 10%), and <1% or not detected for amikacin, ceftolozane/tazobactam and ceftazidime/avibactam, colistin, ertapenem and imipenem/relebactam. Ceftaroline, ceftobiprole and cefuroxime had higher resistance rates than ceftazidime and cefotaxime, partially reflecting the fact that the former agents are better substrates for the K1 chromosomal β-lactamase,^15–17^ which—based on phenotypes—was estimated to be hyperproduced by *c.* 8%–10% of isolates. ESBL prevalence fell from 4.2% in 2001–04 to 0.7% in 2015–19, matching resistance to ceftazidime, which evades K1 enzyme. AmpC β-lactamase production was very rare, and carbapenemases were seen in just two isolates, both from 2017 with *bla*_KPC_.

#### K. aerogenes

Both datasets have limitations for *K. aerogenes*: the BSAC collection comprised only 490 isolates (mean 26/year; see Table S4) whereas frequent co-amoxiclav ‘susceptibility’ during the first 10–12 years suggested that the UKHSA data extract (Figure 3) was substantially compromised by inclusion of *K. pneumoniae* under its earlier informal UK name of ‘*K. aerogenes*.’ (*K. aerogenes,* as now defined, is inherently resistant to co-amoxiclav owing to inducible AmpC.) From 2009 to 2011 the numbers of ‘*K. aerogenes*’ reported to the UKHSA halved, while the prevalence of co-amoxiclav resistance rose from *c.* 40%–50% to >90% (Figure 3b).

Considering only 2015–19, i.e. after the nomenclature was stably resolved, UKHSA surveillance recorded broadly steady prevalence of resistance to third-generation cephalosporins (23%), ciprofloxacin (3%), gentamicin (1%) and imipenem/meropenem (<1%). BSAC surveillance detected AmpC hyperproduction in 27% of isolates, but ESBL production in <1%. One isolate, from 2004, had a carbapenemase gene (*bla*_KPC_).

### Enterobacter *spp.*

*Enterobacter* has undergone multiple taxonomic reorganizations besides the reassignment of ‘*E. aerogenes*’.^18^ These were applied retrospectively to the BSAC data. Isolates identified only to genus level (i.e. as *Enterobacter* spp.), which could not be reclassified, were excluded, along with ‘*E. aerogenes*’. The remaining 3064 isolates all belonged to the *Enterobacter cloacae* complex (see Tables S2 and S7) and these are reviewed.

**Figure 4.**
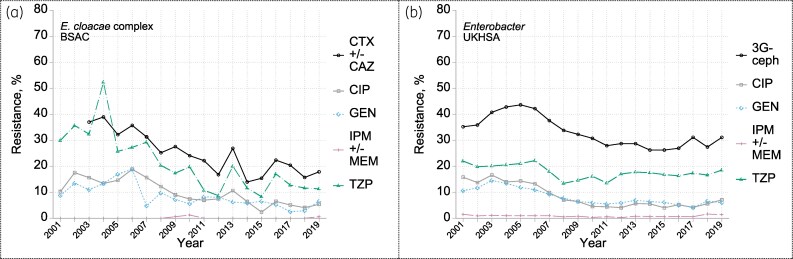
Resistance trends among *Enterobacter* from bacteraemia in (a) BSAC and (b) UKHSA surveillance. Resistance to: CTX +/- CAZ one or both of cefotaxime and ceftazidime; CIP ciprofloxacin; GEN gentamicin; IPM +/- MEM one or both of imipenem and meropenem; TZP piperacillin/tazobactam; 3G-ceph any one or more of CTX, CAZ and (in UKHSA data only and very rarely tested) cefotetan.

Following earlier rises, preceding the present surveillance,^19^ the prevalence of resistances to ciprofloxacin and gentamicin in the UKHSA data fell in parallel, from around 16% in 2003 to near 5% from 2010 (Figure 4). Third-generation cephalosporin resistance also showed a substantial fall, from >40% in 2003–06 to generally <30% after 2011. Piperacillin/tazobactam resistance dipped briefly to just under 15% in 2008–11 between plateaux of *c.* 21% in 2001–06 and 17% in 2012–19. Despite greater year-to-year variability, BSAC data were comparable, albeit that the fall in third-generation cephalosporin resistance was more pronounced and more closely mirrored by piperacillin/tazobactam. Imipenem and meropenem resistances were rare (<2% in both datasets), but ertapenem resistance (BSAC data) averaged 15%—far more than for any other Enterobacterales (see MIC distributions in the Appendix to the Supplementary Data). Mostly this ertapenem resistance was low level, with 61% of MICs for resistant isolates only one dilution above the breakpoint, at 1 mg/L, 28% at 2 mg/L and only 11% at ≥ 4 mg/L (see MIC plots in the Supplementary data).

Among BSAC isolates, resistance to cefotaxime was slightly more prevalent than to ceftazidime and ceftobiprole, with all three resistances largely reflecting AmpC hyperproduction, as indicated by cefotaxime/cloxacillin synergy for *c*. 76% of the cefotaxime-resistant isolates (Figure 2). ESBL production was seen in only 31% of cefotaxime-resistant isolates, including some also with AmpC hyperproduction. Around half of the ESBL producers had *bla*_CTX-M_. Ceftazidime resistance due to ESBL or AmpC was overcome by addition of avibactam, whereas 38% of AmpC hyperproducers (versus 1% of non-hyperproducers) were resistant to ceftolozane/tazobactam (not shown).

Resistance to ciprofloxacin and gentamicin followed similar trends to ESBL production. Tobramycin resistance mirrored gentamicin, but only <1% of isolates were resistant to amikacin. The apparent rise in colistin resistance from a mean of 7% in 2011–14 to 13% in 2015–19 in BSAC data could not be compared with a wider sample as colistin was not included in the UKHSA data extract and is not routinely tested by most hospital laboratories. A previous analysis of BSAC data showed the trait to be associated with particular *E. cloacae* complex genotypes, corresponding to *Enterobacter asburiae*.^20^ Tigecycline had a mode MIC of 0.5 mg/L and no breakpoint (see MIC distributions in the Appendix to the Supplementary Data). Ceftaroline resistance was more prevalent than to cefotaxime, partly reflecting a lower breakpoint. Five carbapenemase producers—one each with *bla*_OXA-48-like_, *bla*_KPC_, *bla*_NDM_, *bla*_IMP_ and *bla*_VIM_—were identified between 2009 and 2019. Three earlier isolates identified only as *Enterobacter* spp., and therefore otherwise excluded from the present analysis, also were resistant to imipenem. One of these, later identified as *Enterobacter cancerogenus*, had *bla*_KPC-4_.

### *Proteeae:* Proteus, Morganella and Providencia *spp.*

Among UKHSA reports for Proteeae bacteraemias, the proportion indicating *Proteus mirabilis* increased from ∼65% to 76% over the 19 years from 2001, balanced by declines in isolates only identified as *Proteus* spp. (down 6 points to 5%), other named *Proteus* (down 2 points to 2%) and *Morganella* (down 3 points to 13%); *Providencia* remained stable at 3%–4% (not shown). By contrast, the proportion of *Morganella* among isolates in the BSAC Proteeae collection collapsed from 14% in 2001–04 to 3% in 2015–19 with smaller reductions for *Providencia* and ‘other’ (non-*mirabilis*) *Proteus*, balanced by the proportion of *P. mirabilis* rising from 79% to 94% (see Table S4). We suspect that labs increasingly misinterpreted ‘Proteeae’ to mean ‘*Proteus*’, excluding other members of the tribe.

**Figure 5.**
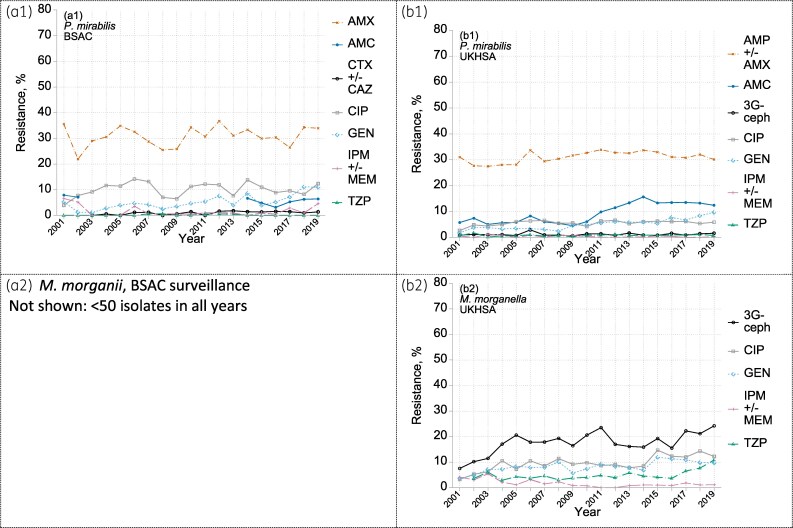
Resistance trends among (a) *P. mirabilis* and (b) *M. morganii* from bacteraemia in (1) BSAC and (2) UKHSA surveillance. Resistance to: AMX amoxicillin; AMC co-amoxiclav; CTX +/- CAZ one or both of cefotaxime and ceftazidime; CIP ciprofloxacin; GEN gentamicin; IPM +/- MEM one or both of imipenem and meropenem; TZP piperacillin/tazobactam; 3G-ceph any one or more of CTX, CAZ and (in UKHSA data only and very rarely tested) cefotetan. BSAC *M. morganii* not shown (≤50 isolates in all years). Breaks in BSAC co-amoxiclav data reflect the period when BSAC tested only the 2:1 amoxicillin:clavulanate combination: the resulting data cannot be analysed against the current breakpoint, which is based on testing with fixed 2 mg/L of clavulanate.

Resistance prevalence among *P. mirabilis* isolates was broadly stable (Figure 5), with similar prevalence recorded in both surveillances for amoxicillin/ampicillin (31%); ciprofloxacin 6% (10% in BSAC) and <2% for carbapenems, third-generation cephalosporins and piperacillin/tazobactam. Resistance to gentamicin at least doubled, mostly after 2010, approaching 10% by 2019. Co-amoxiclav resistance was stable at 6% in BSAC data (comparing the two periods when the combination was tested with a fixed concentration of 2 mg/L clavulanate) but increased suddenly after 2009 in UKHSA reports to a peak of 16% in 2014 and a later plateau of 13%, again likely reflecting changes in testing.

Resistance to additional and newer agents tested in the BSAC surveillance was seldom detected among *P. mirabilis* isolates, except for ceftaroline (6%) (see MIC distributions in the Appendix to the Supplementary Data). Thus, resistance prevalence was <1% for amikacin, tobramycin, ertapenem, ceftobiprole, ceftolozane/tazobactam and ceftazidime/avibactam and <2% for cefuroxime. The mode MIC of tigecycline for BSAC-collected *P. mirabilis* was 4 mg/L, reflecting inherent resistance. The few non-*mirabilis Proteus*, predominantly *P. vulgaris*, were notable for frequent resistance to ceftobiprole (77/105; 73%).

*Morganella morganii*, unlike *P. mirabilis*, is inherently resistant to ampicillin and co-amoxiclav. In addition, UKHSA data showed third-generation cephalosporin resistance rising from around 10% in 2002–03 to steady at around 20% in 2005–19; rates of resistances to cefotaxime and ceftazidime were lower in BSAC data, at around 5%, and only 1% for ceftobiprole (3/265) (see MIC distributions in the Appendix to the Supplementary Data). Derepressed AmpC activity was detected in 8% of BSAC-collected *M. morganii* isolates. Based on UKHSA data, piperacillin/tazobactam resistance was generally near 5%, rising after 2016 to reach 10% in 2019, whereas no such resistance was detected in BSAC isolates (*N* = 377). The UKHSA resistance estimates for piperacillin/tazobactam are surprisingly high, given that tazobactam inhibits *M. morganii* AmpC. Ciprofloxacin and gentamicin resistance stabilized at *c*. 10% after an early rise. No carbapenemases were found in any Proteeae isolate collected.

### Serratia *spp.*

The BSAC surveillance collected *Serratia* in a mixed ‘other Gram-negatives’ group until 2007 (yielding 50–93 isolates/year) and thereafter as a designated group (119–189 isolates/year). The annual collections consistently comprised *c*. 90% *Serratia marcescens* and 10% *Serratia liquefaciens* (see Table S4). Early UKHSA reports (2001–02), based on local identifications, showed proportions of *S. marcescens*, *S. liquefaciens* and unspecified *Serratia* at 63%, 16% and 17%, respectively (not shown). By 2018–19, these proportions had shifted to 88%, 5% and 5%, respectively, resembling the BSAC series.

**Figure 6.**
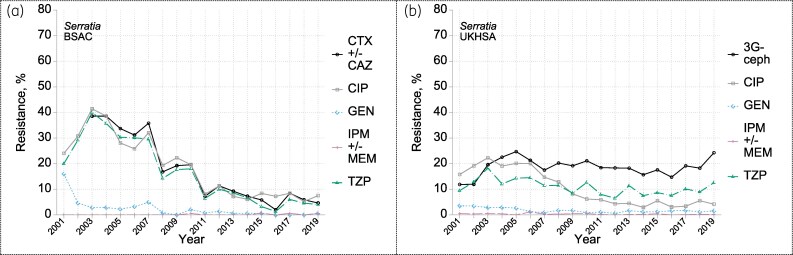
Resistance trends among *Serratia* from bacteraemia in (a) BSAC and (b) UKHSA surveillance. Resistance to: CTX +/- CAZ one or both of cefotaxime and ceftazidime; CIP ciprofloxacin; GEN gentamicin; IPM +/- MEM one or both of imipenem and meropenem; TZP piperacillin/tazobactam; 3G-ceph any one or more of CTX, CAZ and (in UKHSA data only and very rarely tested) cefotetan. In BSAC surveillance, there were fewer *Serratia* isolates/year in 2001–07 (50–93) than in 2008–19 (119–189).

Resistance to ciprofloxacin peaked in 2003 at 22% in UKHSA reports and at 41% in BSAC surveillance, before dropping to level-out near 4% (UKHSA) and 7% (BSAC) by 2012–19 (Figure 6). Proportions resistant to piperacillin/tazobactam, cefotaxime and ceftazidime (also, not shown, ceftobiprole) trended downwards among BSAC isolates, but not for UKHSA reports, where reductions were attenuated (piperacillin/tazobactam) or minimal (third-generation cephalosporins) (Figure 6). This divergence is one of the most striking seen between UKHSA and BSAC data, and we lack good explanation. There was little resistance to gentamicin (overall <2%) or imipenem and/or meropenem (overall <1%) in either dataset.

Based on BSAC testing only, tigecycline (with no breakpoint) had a mode MIC of 1 mg/L. There was widespread low-level resistance to ceftaroline (>50% in 2014 and 2019; mode MIC 1 mg/L). On the other hand, resistance prevalence was below 2% for ertapenem (mode MIC 0.03 mg/L) and <1% for amikacin (mode MIC 2 mg/L), tobramycin (2 mg/L), ceftazidime/avibactam (0.25 mg/L), ceftolozane/tazobactam (0.5 mg/L) and imipenem/relebactam (0.25 mg/L) (see MIC distributions in the Appendix to the Supplementary Data).

ESBLs were rare (<1%). The prevalence of AmpC β-lactamase hyperproduction decreased on the same timescale as cefotaxime resistance, although (in contrast to e.g. *Enterobacter*) not all AmpC producers were resistant to both cefotaxime and ceftazidime. Thus, in 2012–19, 36 of 141 AmpC hyperproducers counted as susceptible to cefotaxime and 25 were ‘susceptible, increased exposure’. AmpC hyperproduction was less prevalent in *S. liquefaciens* (4%) than *S. marcescens* (18%) with related resistances correspondingly less prevalent (not shown). Carbapenemase genes were found in three *S. marcescens—*two with *bla*_OXA-48-like_ from 2015 and 2017, and one with *bla*_NDM_ from 2019.

### Pseudomonas

*Pseudomonas aeruginosa* comprised 95% (4015/4228) of *Pseudomonas* isolates collected in the BSAC surveillance, ranging between 92% and 99% by year without discernible trend (see Table S4). Sixteen other species were recorded, with *Pseudomonas putida* (59, 1.4%), *Pseudomonas fluorescens* (44, 1.0%) and *Pseudomonas stutzeri* (41, 1.0%) the most frequent. In the UKHSA surveillance, a diminishing minority of isolates (15% in 2001, 8% in 2019) were not identified to species level; *P. aeruginosa* comprised a stable 88%–91% among those identified, with the confounder that some uncommon former *Pseudomonas* spp. have gradually been moved to other genera,^21^ slightly distorting the denominator.

**Figure 7.**
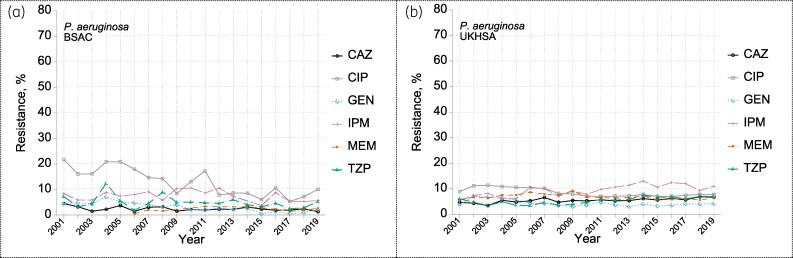
Resistance trends among *P. aeruginosa* from bacteraemia in (a) BSAC and (b) UKHSA surveillance. Resistance to: CAZ ceftazidime; CIP ciprofloxacin; GEN gentamicin; IPM imipenem; MEM meropenem; TZP piperacillin/tazobactam.

Resistance rates for *P. aeruginosa* largely remained <10% for ceftazidime, gentamicin, meropenem and piperacillin/tazobactam in both surveillances (Figure 7). A fall in ciprofloxacin resistance in the BSAC data, from 16–22% in 2001–04 to 6–10% in 2015–19, was replicated in attenuated form in the UKHSA data. UKHSA data suggested a small rise in imipenem resistance, from around 7% in the first few years to around 11% in the last few; the BSAC surveillance lacked power to detect such a small shift.

The prevalence of resistance in *P. aeruginosa* was <2% for each of six additional antimicrobials tested intermittently in the BSAC surveillance: amikacin (mode MIC, 2 mg/L), ceftazidime/avibactam (2 mg/L), ceftolozane/tazobactam (0.5 mg/L), colistin (1 mg/L), imipenem/relebactam (0.25 mg/L) and tobramycin (0.25 mg/L). Ceftobiprole, with no breakpoint, had a mode MIC of 2 mg/L (see MIC distributions in the Appendix to the Supplementary Data).

Resistance mechanisms were sought according to fixed protocols in the second decade of the BSAC surveillance—earlier examination was *ad hoc*, concentrating on MIC profiles deemed ‘unusual’. Our best estimates of prevalence for *P. aeruginosa*, based on different periods when reliable estimation was possible, were 2% for AmpC hyperproduction (19/919; 2014 and 2017–19), 6% for OprD deficiency (125/1962; 2011–19) and 5% for up-regulated efflux (67/1235; 2014–19). There were sporadic detections of MBL genes between 2012 and 2019—four in *P. aeruginosa* (three *bla*_VIM_ and one *bla*_NDM_) and one in *P. putida* (*bla*_VIM_), without temporal patterns.

## Discussion

During the BSAC surveillance’s two decades, global concern about resistance increasingly migrated to Gram-negative opportunists. Carbapenem-resistant and ESBL-producing Enterobacterales top the WHO’s list of antibiotic resistance concerns, along with carbapenem-resistant *Acinetobacter baumannii.*^22^ UK experience partly supports these priorities, but also reveals nuanced patterns of rising and falling resistance.

Both the BSAC (UK and Ireland) and the UKHSA (England only) surveillances found increasing resistance to cephalosporins, fluoroquinolones and gentamicin among *E. coli* and *Klebsiella* spp. early in the present century. These shifts were described contemporaneously and, for *E. coli*, reflected import and spread of CTX-M ESBLs, often carried by the international ST131 lineage.^23,24^ By the mid-2010s, ST131 accounted for 64% of bloodstream ESBL *E. coli* in the UK.^23^ Nevertheless, these early rises stalled after 2007 and were followed by stabilization or declines for *E. coli* and *K. pneumoniae.*^25^ Rises resumed only recently, principally for *K. pneumoniae*, though with rates still below 20% in 2019.

There was less evidence to support concern apropos other Enterobacterales. For *P. mirabilis*, resistance was uncommon and stable for ciprofloxacin and cephalosporins, although that to gentamicin roughly doubled, mostly after 2010. For *Enterobacter* and *Serratia* spp., cephalosporin and fluoroquinolone resistance trended downwards, with the former decline reflecting a diminishing tally of AmpC hyperproducers. Declining resistance in *Serratia* was more evident in BSAC than UKHSA data, which for cephalosporins (not fluoroquinolones), may be distorted by some hospitals reporting all AmpC-inducible species, including serratias, as resistant. For bloodstream *P. aeruginosa*, resistance rates remained low throughout, extending the pattern of UK *P. aeruginosa* surveys from the 1980s onwards.^26^

Despite concerns predicated upon cases, colonizations and outbreaks, Enterobacterales with carbapenemases remained extremely rare; the BSAC collected few producers and UKHSA surveillance shows imipenem and meropenem resistance rates of 1%–2% for *K. pneumoniae* and <1% for other Enterobacterales.

EARS-net bacteraemia surveillance shows that many continental EU/EEA countries have more prevalent resistance, especially among bloodstream *K. pneumoniae*, where post-2015 rates for third-generation cephalosporins exceeded 25% everywhere except Germany, the Netherlands and Scandinavia, and topped 50% across eastern Europe.^7^ For fluoroquinolones, resistance rates among bloodstream *K. pneumoniae*, were 4%–14% in Germany, the Netherlands and Scandinavia—resembling the UK—but were vastly higher in Italy (55%) and eastern Europe (*c.* 75%). Carbapenem resistance rates remained <2% for *K. pneumoniae* across NW Europe, as in the UK and Ireland, but exceeded 25% in Italy and the Balkans, with multiple countries experiencing dramatic rises over brief (2- to 5-year) periods. For bloodstream *E. coli*, European cephalosporin resistance rates were mostly 10%–25% in 2019—similar to the those found here—but were lower in Scandinavia and higher in Southern and Eastern Europe. Carbapenem resistance remained rare everywhere in Europe for *E. coli*. Irrespective of antibiotic, resistance in bloodstream *P. aeruginosa* rises as one moves south and east across Europe, being lowest in Scandinavia and highest in the Balkans.^7^

A plausible explanation for the stalling of cephalosporin and fluoroquinolone resistance in the UK is reduced use of these drugs. By 2019, their in-hospital use comprised just 40% and 78%, respectively, of EU/EEA averages.^27^ This reflects earlier UK shifts, predicated on concern about their roles in selecting colonic overgrowth by toxigenic *Clostridioides difficile*.^28^ Reduced cephalosporin use provides a particularly attractive explanation for reduced resistance in *Enterobacter* and *Serratia* spp., given that most cephalosporin resistance in these genera involves *de novo* selection of AmpC-derepressed mutants.^25^ Accordingly, less selection pressure might be expected to translate directly into less resistance. The smaller and briefer reduction in cephalosporin and fluoroquinolone resistance among *E. coli* likely reflects the very different situation of difficult-to-lose plasmids associated with a globally competitive lineage (ST131).^9,23^

The counterbalance to the UK’s shift away from cephalosporins and fluoroquinolones is increased use of β-lactam/β-lactamase inhibitor combinations. By 2019, UK hospital prescribing of these was 178% of the EU/EEA average.^27^ This raises concerns. First, rates of resistance to co-amoxiclav are extremely high for *E. coli* and rose over time in *K. pneumoniae*, while other important Gram-negative pathogens are inherently resistant. Second, rates of resistance to piperacillin/tazobactam in *K. pneumoniae* resembled those for cephalosporins and fluoroquinolones, despite piperacillin/tazobactam being widely perceived as more active. Third, β-lactam/β-lactamase inhibitor combinations are hard to test reliably, as evidenced by: (i) the difficulties of distinguishing piperacillin/tazobactam susceptibility and resistance in the MERINO trial,^29^ (ii) the greater ‘noise’ on the resistance trend lines for piperacillin/tazobactam than, for example, cephalosporins and quinolones (Figures 1 and 2; see also Allen *et al*.^12^), and (iii) EUCAST now applying an ‘area of technical uncertainty’ for 5/8 β-lactam/β-lactamase inhibitor combinations against Enterobacterales, including co-amoxiclav and piperacillin/tazobactam, as against for just 1/30 single-agent β-lactams.^30^ Co-amoxiclav and piperacillin/tazobactam are valuable antibiotics, but it is disturbing that the UK depends so heavily on combinations that are difficult to test.

Although resistance rates for piperacillin/tazobactam, cephalosporins and fluoroquinolones for Enterobacterales mostly were below 20%, they often exceeded 10%. Such rates are not trivial, translating into >10% risk that empirical therapy will prove ineffective, sometimes fatally.^31^ A promising route to improvement lies in faster diagnostics, allowing earlier tailoring of therapy. It is now commonplace for laboratories to identify bacteria directly from blood cultures by MALDI-TOF and to perform PCR for MRSA, giving early treatment guidance. Rapid testing of early growth from blood cultures, by disc,^32^ automated microscopy^33^ or molecular methods^34^ can deliver susceptibility predictions within *c*. 24–30 h of blood being drawn; molecular testing directly from blood is challenging but potentially much faster.^35,36^

**Figure 8.**
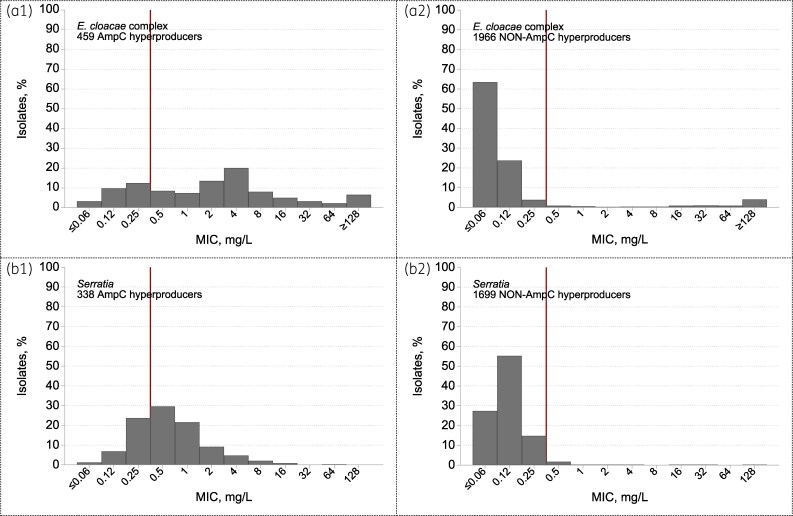
Ceftobiprole MICs for (a) *Enterobacter* and (b) *Serratia* isolates with (1) or without (2) AmpC hyperproduction from BSAC bacteraemia surveillance. The red vertical line marks the EUCAST resistance breakpoint (*R* > 0.25 mg/L). Seventy-five percent (342/459) of *Enterobacter* identified as AmpC hyperproducers were resistant to ceftobiprole (MIC > 0.25). Adding together the 25% susceptible (MICs ≤ 0.25 mg/L) and the 16% with MICs at 0.5 or 1 mg/L (up to two dilutions above the *R* breakpoint), a total of 41% were inhibited by concentrations ≤ 1 mg/L. Sixty-eight percent (231/338) of *Serratia* identified as AmpC hyperproducers were resistant to ceftobiprole (MIC  > 0.25). Adding together the 32% susceptible (MIC  ≤ 0.25 mg/L) and the 51% with MICs at 0.5 or 1 mg/L (up to 2 dilutions above the R breakpoint), a total of 83% were inhibited by concentrations ≤ 1 mg/L.

Several novel agents were tested in the BSAC Programme, with many results already published. Ceftazidime/avibactam was active against almost all ESBL-producing and AmpC-hyperproducing Enterobacterales, also those with serine carbapenemases (except one *Serratia* with an OXA-48-like enzyme), although not those with MBLs. Ceftolozane/tazobactam proved the most-widely-active agent after colistin against *P. aeruginosa*, retaining activity against the great majority of isolates with efflux-, AmpC- and OprD-related resistance.^37^ Activity was lost against *P. aeruginosa* with MBLs. Ceftolozane/tazobactam also was widely active against ESBL-producing Enterobacterales although less consistently so than carbapenems, particularly for *K. pneumoniae*.^37^ Imipenem/relebactam was active against most imipenem-resistant *P. aeruginosa*, reflecting the fact that their ‘impermeability-type resistance’, contingent on loss of OprD, co-depends on expression of the relebactam-inhibited AmpC chromosomal β-lactamase.^38^ The few *P. aeruginosa* with MBLs were resistant. Imipenem/relebactam inhibits KPC carbapenemases but was not tested in the years when producers were collected. Ceftaroline and ceftobiprole are of greater interest for their anti-MRSA activity, but were tested against Gram-negatives, the former in 3 years and the latter for 15. Both are challenged by low breakpoints.^30^ In general, ceftaroline lost activity when Enterobacterales had ESBLs or hyperproduced AmpC, with some resistance also in isolates lacking these enzymes, confirming prior reults^39^ it lacks anti-*Pseudomona*s activity. Ceftobiprole is more complex. It lacked activity against ESBL producers in general and *K. oxytoca* inferred to hyperproduce K1 enzyme; MICs for AmpC-derepressed isolates were lower than for example cefotaxime, but displayed a very splayed distribution with 75% of AmpC-hyperproducing *Enterobacter* resistant at breakpoint (Figure 8). Its MICs were low for many *P. aeruginosa* (mode, 2 mg/L), but EUCAST cites ‘insufficient evidence’ for a clinical breakpoint.^30^ Tigecycline—widely tested in the BSAC Programme’s earlier years (2004–13)—had low MICs for many Enterobacterales, including multiresistant organisms, but EUCAST now only has breakpoints for *E. coli*. For all species, it showed tight MIC distributions without evidence of acquired resistance.

In summary, and except for co-amoxiclav, the rates of resistance among Gram-negative bloodstream pathogens found in these surveillances, spanning almost 20 years, remain low by European standards, especially for *Klebsiella* spp. Newer compounds—ceftazidime/avibactam, ceftolozane/tazobactam and imipenem/relebactam—overcome most, but not all, resistance types, as do further molecules and combinations, e.g. meropenem/vaborbactam and cefiderocol that were not tested. These newer agents have more predictable pharmacokinetics and cause far less toxicity than polymyxins, which, accordingly, the Infectious Disease Society of America argues they should replace wherever microbiologically warranted.^40^

Concerns nonetheless remain. First, experience with cephalosporins and fluoroquinolones from 2002 to 2006 shows how swiftly resistance can rise in *E. coli*—the most frequent agent of bacteraemia.^9^ Carbapenemase producers may disseminate similarly in the future. This hazard increases when resistance establishes in the community—as has already happened with *E. coli* producing NDM carbapenemases in India (and likely other parts of the Indian subcontinent),^41^ a region with which the UK enjoys considerable population exchanges. Second, although resistance rates are low in absolute terms, they are sufficient to present a risk that empirical therapy may fail in a significant minority of patients. This encourages empirical carbapenem use and polypharmacy.^40^ Better solutions, from a stewardship perspective, lie in accelerated susceptibility testing, which also may identify patients in whom otherwise relegated antibiotics (e.g. co-trimoxazole, trimethoprim and chloramphenicol) might still be used. Such re-adoption would diminish selection pressure on current standard-of-care agents. Third, there must be concern about the extent to which the UK hospital therapy depends on penicillin/β-lactamase inhibitor combinations given prevalent co-amoxiclav resistance and the susceptibility testing challenges. Lastly, Gram-negative bacteria have repeatedly surprised with their adaptability, as recently with the emergence of PBP3-associated resistance to multiple β-lactams in *E. coli*.^42^ Moreover, while resistance rates have not increased dramatically, numbers of Gram-negative bacteraemias reported in the UK rose steadily to 2019, particularly for *E. coli*, increasing the societal burden of resistance.^3^ All this underscores the need for continued surveillance.

## Supplementary Material

dkaf250_Supplementary_Data

## Transparency declarations

S.M., R.H., R.A., A.C. and O.N. are employees of the UKHSA. R.H. and O.N. work for the Antimicrobial Resistance and Healthcare Associated Infections Division and have no conflict of interest to declare. S.M., A.C. and R.A. work for the Antimicrobial Resistance and Healthcare Associated Infections Reference Unit (AMRHAI), which has received financial support for conference attendance, research projects or contracted evaluations from numerous sources, including Accelerate Diagnostics, Allecra Therapeutics, AstraZeneca UK Ltd, AusDiagnostics, Basilea Pharmaceutica, Becton Dickinson Diagnostics, bioMérieux, Bio-Rad Laboratories, BSAC, Cepheid, Check-Points B.V., UK Department of Health, Enigma Diagnostics, Food Standards Agency, GlaxoSmithKline Services Ltd, Helperby Therapeutics, Henry Stewart Talks, IHMA Ltd, Innovate UK, Integra holdings, Merck Sharpe & Dohme Corp., Meiji Seika Pharma Co. Ltd, Mobidiag, Momentum Biosciences Ltd, Neem Biotech, Nordic Pharma Ltd, Norgine Pharmaceuticals, Paratek Pharmaceuticals, Roche, Smith & Nephew UK Ltd, Shionogi & Co. Ltd, Trius Therapeutics, VenatoRx Pharmaceuticals and Wockhardt Ltd. C.H. was employed by Una Health Limited and QuantuMDx Group Limited during the period of data analysis and manuscript preparation. M.A. is a Trustee of the BSAC Council and is employed by MSD (UK) Limited, London, UK. M.A. has shareholdings in Merck & Co., Inc., Rahway, NJ, USA. C.L. is a Trustee of the BSAC Council and is employed by Shionogi B.V. B.J.P.: consultancy: Genpax. D.M.L.: advisory boards or *ad hoc* consultancy: AdjuTec, AstraZeneca, Centauri, Genpax, GSK, LipoVation, Meiji, Paion, Shionogi, Sumitovant, Thermo Fisher and Wockhardt; paid lectures: bioMérieux, Pfizer, Shionogi and Zuellig Pharma; and relevant shareholdings or options: Genpax, GSK, Merck & Co. Inc., and Revvity amounting to <10% of portfolio value. D.M.L. also has shareholdings in Arecor, GENinCode, Probiotix, Rua Life Sciences, SkinBioTherapeutics and VericiDx (all with research/products pertinent to medicines or diagnostics) through Enterprise Investment Schemes but has no authority to trade these holdings directly. All other authors have nothing to declare.

## Supplementary data

Supplementary Figures S1–S5 and Tables S1–S15 and Appendix are available as Supplementary data at *JAC* Online.

## References

[1] Bryan CS . Clinical implications of positive blood cultures. Clin Microbiol Rev 1989; 2: 329–53. 10.1128/CMR.2.4.329 2680055 PMC358128

[2] Wilson J, Elgohari S, Livermore DM et al Trends among pathogens reported as causing bacteraemia in England, 2004–2008. Clin Microbiol Infect 2011; 17: 451–8. 10.1111/j.1469-0691.2010.03262.x 20491834

[3] *English Surveillance Programme for Antimicrobial Utilisation and Resistance (ESPAUR) Report 2020 to 2021*. 2020. https://webarchive.nationalarchives.gov.uk/ukgwa/20221020175458/https://www.gov.uk/government/publications/english-surveillance-programme-antimicrobial-utilisation-and-resistance-espaur-report.

[4] Aliabadi S, Jauneikaite E, Müller-Pebody B et al Exploring temporal trends and risk factors for resistance in *Escherichia coli*-causing bacteraemia in England between 2013 and 2018: an ecological study. J Antimicrob Chemother 2022; 77: 782–92. 10.1093/jac/dkab440 34921311

[5] Maher C, Hassan KA. The Gram-negative permeability barrier: tipping the balance of the in and the out. mBio 2023; 14: e0120523. 10.1128/mbio.01205-23 37861328 PMC10746187

[6] Jacoby GA . Ampc β-lactamases. Clin Microbiol Rev 2009; 22: 161–82. 10.1128/CMR.00036-08 PMC2620637 19136439

[7] Anon . European Antimicrobial Resistance Surveillance Network (EARS-net). Eur Cent Dis Prev Control. https://www.ecdc.europa.eu/en/about-us/partnerships-and-networks/disease-and-laboratory-networks/ears-net.

[8] Fuzi M, Rodriguez Baño J, Toth A. Global evolution of pathogenic bacteria with extensive use of fluoroquinolone agents. Front Microbiol 2020; 11: 271. 10.3389/fmicb.2020.00271 32158437 PMC7052298

[9] Nicolas-Chanoine M-H, Bertrand X, Madec J-Y. *Escherichia coli* ST131, an intriguing clonal group. Clin Microbiol Rev 2014; 27: 543–74. 10.1128/CMR.00125-13 24982321 PMC4135899

[10] Livermore DM, Canton R, Gniadkowski M et al CTX-M: changing the face of ESBLs in Europe. J Antimicrob Chemother 2007; 59: 165–74. 10.1093/jac/dkl483 17158117

[11] van Duin D, Doi Y. The global epidemiology of carbapenemase-producing Enterobacteriaceae. Virulence 2017; 8: 460–9. 10.1080/21505594.2016.1222343 27593176 PMC5477705

[12] Allen M, Reynolds R, Mushtaq S et al The British Society for Antimicrobial Chemotherapy Resistance Surveillance Project: methods and limitations. J Antimicrob Chemother 2025; 80 (Suppl 4): iv7–iv21. 10.1093/jac/dkn348 18819978

[13] Bischoff D . BLINDSCHEMES: Stata module to provide graph schemes sensitive to color vision deficiency,” Statistical Software Components S458251, Boston College Department of Economics, revised 07 Aug 2020. https://ideas.repec.org/cgi-bin/refs.cgi.

[14] Reynolds R, Mushtaq S, Allen MK et al Impact of changed co-amoxiclav susceptibility testing formats on apparent resistance rates for bloodstream *Escherichia coli* in a long-term surveillance. J Antimicrob Chemother 2022; 77: 1206–8. 10.1093/jac/dkab483 35045166

[15] Livermore DM . β-Lactamases in laboratory and clinical resistance. Clin Microbiol Rev 1995; 8: 557–84. 10.1128/CMR.8.4.557 8665470 PMC172876

[16] Livermore DM, Hope R, Brick G et al Non-susceptibility trends among Enterobacteriaceae from bacteraemias in the UK and Ireland, 2001–06. J Antimicrob Chemother 2008; 62: ii41–54. 10.1093/jac/dkn351 18819979

[17] Mushtaq S, Warner M, Williams G et al Activity of chequerboard combinations of ceftaroline and NXL104 versus β-lactamase-producing Enterobacteriaceae. J Antimicrob Chemother 2010; 65: 1428–32. 10.1093/jac/dkq161 20478991

[18] Davin-Regli A, Lavigne J-P, Pagès J-M. *Enterobacter* spp.: update on taxonomy, clinical aspects, and emerging antimicrobial resistance. Clin Microbiol Rev 2019; 32: e00002–19. 10.1128/CMR.00002-19 31315895 PMC6750132

[19] Livermore DM, James D, Reacher M et al Trends in fluoroquinolone (ciprofloxacin) resistance in Enterobacteriaceae from bacteremias, England and Wales, 1990–1999. Emerg Infect Dis 2002; 8: 473–8. 10.3201/eid0805.010204 11996681 PMC2732494

[20] Mushtaq S, Reynolds R, Gilmore MC et al Inherent colistin resistance in genogroups of the *Enterobacter cloacae* complex: epidemiological, genetic and biochemical analysis from the BSAC Resistance Surveillance Programme. J Antimicrob Chemother 2020; 75: 2452–61. 10.1093/jac/dkaa201 32514538

[21] Lalucat J, Gomila M, Mulet M et al Past, present and future of the boundaries of the *Pseudomonas* genus: proposal of *Stutzerimonas* gen. Nov. Syst Appl Microbiol 2022; 45: 126289. 10.1016/j.syapm.2021.126289 34920232

[22] Anon . WHO updates list of drug-resistant bacteria most threatening to human health. https://www.who.int/news/item/17-05-2024-who-updates-list-of-drug-resistant-bacteria-most-threatening-to-human-health.

[23] Day MJ, Hopkins KL, Wareham DW et al Extended-spectrum β-lactamase-producing *Escherichia coli* in human-derived and foodchain-derived samples from England, Wales, and Scotland: an epidemiological surveillance and typing study. Lancet Infect Dis 2019; 19: 1325–35. 10.1016/S1473-3099(19)30273-7 31653524

[24] Livermore DM, Hawkey PM. CTX-M: changing the face of ESBLs in the UK. J Antimicrob Chemother 2005; 56: 451–4. 10.1093/jac/dki239 16006451

[25] Livermore DM, Hope R, Reynolds R et al Declining cephalosporin and fluoroquinolone non-susceptibility among bloodstream Enterobacteriaceae from the UK: links to prescribing change? J Antimicrob Chemother 2013; 68: 2667–74. 10.1093/jac/dkt212 23766490

[26] Henwood CJ, Livermore DM, James D et al Antimicrobial susceptibility of *Pseudomonas aeruginosa*: results of a UK survey and evaluation of the British Society for Antimicrobial Chemotherapy disc susceptibility test. J Antimicrob Chemother 2001; 47: 789–99. 10.1093/jac/47.6.789 11389111

[27] Anon . Antimicrobial consumption—annual epidemiological report for 2019. 2020. https://www.ecdc.europa.eu/en/publications-data/surveillance-antimicrobial-consumption-europe-2019.

[28] Dingle KE, Didelot X, Quan TP et al Effects of control interventions on *Clostridium difficile* infection in England: an observational study. Lancet Infect Dis 2017; 17: 411–21. 10.1016/S1473-3099(16)30514-X 28130063 PMC5368411

[29] Henderson A, Paterson DL, Chatfield MD et al Association between minimum inhibitory concentration, β-lactamase genes and mortality for patients treated with piperacillin/tazobactam or meropenem from the MERINO study. Clin Infect Dis 2021; 73: e3842–50. 10.1093/cid/ciaa1479 33106863

[30] Anon . EUCAST: Clinical breakpoints and dosing of antibiotics. https://www.eucast.org/clinical_breakpoints/.

[31] Zasowski EJ, Bassetti M, Blasi F et al A systematic review of the effect of delayed appropriate antibiotic treatment on the outcomes of patients with severe bacterial infections. Chest 2020; 158: 929–38. 10.1016/j.chest.2020.03.087 32446623

[32] Jonasson E, Matuschek E, Kahlmeter G. The EUCAST rapid disc diffusion method for antimicrobial susceptibility testing directly from positive blood culture bottles. J Antimicrob Chemother 2020; 75: 968–78. 10.1093/jac/dkz548 32016342 PMC7069491

[33] Cenci E, Paggi R, Socio GVD et al Accelerate Pheno™ blood culture detection system: a literature review. Future Microbiol 2020; 15: 1595–605. 10.2217/fmb-2020-0177 33215528

[34] Dunbar SA, Gardner C, Das S. Diagnosis and management of bloodstream infections with rapid, multiplexed molecular assays. Front Cell Infect Microbiol 2022; 12: 859935. 10.3389/fcimb.2022.859935 35372128 PMC8966137

[35] Samuel L . Direct-from-blood detection of pathogens: a review of technology and challenges. J Clin Microbiol 2023; 61: e00231-21. 10.1128/jcm.00231-21 37222587 PMC10358183

[36] MacVane SH, Dwivedi HP. Evaluating the impact of rapid antimicrobial susceptibility testing for bloodstream infections: a review of actionability, antibiotic use and patient outcome metrics. J Antimicrob Chemother 2024; 79: i13–25. 10.1093/jac/dkae282 39298359 PMC11412245

[37] Livermore DM, Mushtaq S, Meunier D et al Activity of ceftolozane/tazobactam against surveillance and ‘problem’ Enterobacteriaceae, *Pseudomonas aeruginosa* and non-fermenters from the British Isles. J Antimicrob Chemother 2017; 72: 2278–89. 10.1093/jac/dkx136 28520867 PMC5890766

[38] Horner C, Mushtaq S, Livermore DM et al Potentiation of imipenem by relebactam for *Pseudomonas aeruginosa* from bacteraemia and respiratory infections. J Antimicrob Chemother 2019; 74: 1940–4. 10.1093/jac/dkz133 31032858

[39] Mushtaq S, Warner M, Ge Y et al *In vitro* activity of ceftaroline (PPI-0903M, T-91825) against bacteria with defined resistance mechanisms and phenotypes. J Antimicrob Chemother 2007; 60: 300–11. 10.1093/jac/dkm150 17548456

[40] Tamma PD, Aitken SL, Bonomo RA et al Infectious Diseases Society of America guidance on the treatment of AmpC β-lactamase-producing Enterobacterales, carbapenem-resistant *Acinetobacter baumannii*, and *Stenotrophomonas maltophilia* infections. Clin Infect Dis 2022; 74: 2089–114. 10.1093/cid/ciab1013 34864936

[41] Arum N, Ghafur A, Kazi M et al Prevalence of faecal carriage of carbapenemase producing Enterobacteriaceae in healthy Indian subjects from the community. Indian J Med Microbiol 2022; 40: 374–7. 10.1016/j.ijmmb.2022.05.010 35691752

[42] Alm RA, Johnstone MR, Lahiri SD. Characterization of *Escherichia coli* NDM isolates with decreased susceptibility to aztreonam/avibactam: role of a novel insertion in PBP3. J Antimicrob Chemother 2015; 70: 1420–8. 10.1093/jac/dku568 25634992

