# Supplementary material for: Antimicrobial resistance among Gram-negative agents of bacteraemia in the UK and Ireland: trends from 2001 to 2019
Source: J Antimicrob Chemother. 2025 Oct 27;80(Suppl 4):iv36–48. doi: 10.1093/jac/dkaf250 (PMC12556499; doi:10.1093/jac/dkaf250)

Antimicrobial resistance among Gram-negative agents of bacteraemia in the UK and Ireland: trends from 2001 to 2019

SUPPLEMENTARY INFORMATION

Methods for the BSAC Resistance Surveillance Project are described in detail in a companion paper [BASIL2_Methods][1]. This details also the UKHSA bacteraemia surveillance, to which the BSAC data are cross-related.[2,3] Breakpoints and ECOFFs (Epidemiological Cut-offs) listed in Tables S5–S10 are from EUCAST tables of breakpoints (v12.0) and related guidance at the time of analysis (https://www.eucast.org), specifically:

> European Committee on Antimicrobial Susceptibility Testing. **Breakpoint tables** for interpretation of MICs and zone diameters. Version 12.0, valid from 2022-01-01.". https://www.eucast.org; https://www.eucast.org/fileadmin/src/media/PDFs/EUCAST_files/Breakpoint_tables/v_12.0_Breakpoint_Tables.pdf. Accessed 20 January 2025
>
> European Committee on Antimicrobial Susceptibility Testing. Guidance document: **EUCAST breakpoints in brackets** 1 December, 2021. Available at: https://www.eucast.org/fileadmin/src/media/PDFs/EUCAST_files/Guidance_documents/Breakpoints_in_brackets.pdf /. Accessed 13 January 2025.
>
> European Committee on Antimicrobial Susceptibility Testing. Data from the EUCAST MIC distribution website. https://www.eucast.org/**mic_and_zone_distributions_and_ecoffs**; https://mic.eucast.org/; https://mic.eucast.org/search/.

Contents

BSAC bacteraemia resistance surveillance – isolate collection and testing 3

Table S1. Isolate collection quotas and targets – BSAC Gram-negative bacteraemia surveillance *3*

Table S2. Actual numbers of Gram-negative isolates tested, and centres contributing by year – BSAC Gram-negative bacteraemia surveillance *3*

Table S3. Antimicrobial data excluded after collection, based on evidence of flawed testing (BSAC surveillance) 4

Table S4. Numbers of Gram-negative isolates tested by organism subgroup and year (BSAC surveillance) 5

Table S5. *E. coli*: antibiotics analysed – years included, N of isolates, resistance breakpoints and mode MICs (BSAC surveillance) 6

Table S6. Klebsiellas: antibiotics analysed – years included, N of isolates, resistance breakpoints and mode MIC (BSAC surveillance) 7

Table S7. *E. cloacae* complex: antibiotics analysed – years included, N of isolates, resistance breakpoints and mode MIC (BSAC surveillance) 8

Table S8. Proteeae: antibiotics analysed – years included, N of isolates, resistance breakpoints and mode MIC (BSAC surveillance) 9

Table S9. *Serratia*: antibiotics analysed – years included, N of isolates, resistance breakpoints and

mode MIC (BSAC surveillance) 10
Table S10. *Pseudomonas*: antibiotics analysed – years included, N of isolates, breakpoints and mode MIC (BSAC surveillance) 11
BSAC bacteraemia resistance surveillance – patient characteristics 12
Table S11. Proportion (%) of male patients by organism species 12
Table S12. Patient age: summary measures by organism group 13
Figure S1. Patient age: histograms and trends by organism group 13
Figure S2. Trends in care setting by organism group 15
Table S13. Proportion of isolates from ICU patients, by organism group 16
Figure S3. Trends in proportion of isolates from ICU patients by organism group 16
Tables S14. Source of infection: top three or ≥10% of isolates, by organism 17
UKHSA voluntary bacteraemia surveillance – routine data, England only 18
Table S15. Number of reported Gram-negative bacteraemias, by pathogen group (UKHSA surveillance, routine data) 18
Figure S4. Number of reported Gram-negative bacteraemias (UKHSA surveillance, routine data) 19
Figure S5. Trends in percentage of bacteraemia episodes reported to UKHSA with antimicrobial susceptibility test results, by organism group (UKHSA surveillance) 19
References 21
APPENDIX – MIC distributions 22
E. coli 22
Klebsiella spp. 24
E. cloacae complex 27
P. mirabilis 28
M. morganii 30
Serratia 31
P. aeruginosa 34

BSAC bacteraemia resistance surveillance – isolate collection and testing

Table S1. Isolate collection quotas and targets – BSAC Gram-negative bacteraemia surveillance

Annual collection periods[1]	Target N of centres	*E. coli*		All other collection groups[2]	
		Quota/lab	Target	Quota/lab	Target
2001–2007	25	10	250	10	250
2008–2009	25	20	500	10	250
2010–2015	40	14	560	7	280
2016–2019	25	20	500	10	250

[1] January–December
[2] *Klebsiella* spp., *Enterobacter* spp., Proteeae, *Pseudomonas spp.*, other Gram-negative bacteria (2001–07) / *Serratia* spp. (2008–19). (*Serratia* were collected as part of a mixed "other Gram-negative bacteria" group until 2007, and as a single genus group thereafter).

Collections per participating centre per year averaged >90% of the requested quota of Gram-negative isolates for *E. coli* (97%) and *Klebsiella* spp. (95%), 86–87% for *Pseudomonas* spp. and Proteeae, 76% for *Enterobacter* spp. and 67% for the other Gram-negatives/*Serratia* spp. group.

Table S2. Actual numbers of Gram-negative isolates tested, and centres contributing by year – BSAC Gram-negative bacteraemia surveillance

Year	N of Centres (actual)[1]	N of isolates					
		E. coli	*Klebsiella*[2]	*E. cloacae* complex[3]	Proteeae	*Serratia*[4]	*Pseudo-monas* spp.
2001	24	245	255	137	202	50	196
2002	25	250	264	171	203	65	196
2003	25	248	267	173	232	70	222
2004	25	248	248	172	217	70	234
2005	25	247	252	171	222	89	226
2006	25	242	255	154	215	93	217
2007	25	248	259	147	205	81	211
2008	24	467	220	123	189	119	192
2009	25	469	242	167	209	130	212
2010	39	531	295	162	257	189	251
2011	38	522	270	158	229	124	218
2012	38	520	287	161	214	150	231
2013	39	539	310	160	246	152	242
2014	40	547	296	172	237	165	238
2015	40	548	304	169	243	155	227
2016	25	496	273	170	207	152	234
2017	25	477	260	157	218	166	222
2018	24	475	266	172	216	153	221
2019	24	477	285	168	231	173	238
Total	(80)	7796	5108	3064	4192	2346	4228

See Table S4 for species within these genera and groups e.g. *K. oxytoca, P. aeruginosa.*
Numbers are shown after reclassifying isolates retrospectively where required by changes in taxonomy during the course of the surveillance.
[1] Number of sites that actually contributed any Gram-negative isolates from bacteraemia in that year; not all necessarily contributed isolates of all organism groups.
[2] The *Klebsiella* collection includes 478 isolates collected in 2001–2018 as *E. aerogenes* and subsequently reclassified.

[3] *Enterobacter cloacae* complex only. Isolates collected as *E. aerogenes* (N=478) were reclassified to *Klebsiella*; other isolates collected as *Enterobacter* and reclassified to other genera (40 *Cronobacter*, 8 *Lelliottia*, 4 *Pluralibacter*) were excluded and are not shown; no other *Enterobacter* isolates were identified on receipt as species outside the *E. cloacae* complex. A random sample of *Enterobacter* isolates recorded without a species identification (2001–2010) were retested by MALDI-TOF in 2022; the majority (20/25) were *E. cloacae* complex and were included in analysis; the remaining five (which included genera other than *Enterobacter*), and 136 other isolates identified only as *Enterobacter* spp. (and not retested) were excluded from analysis and are not shown.
[3,4] *Serratia* were collected as part of a mixed "other Gram-negative bacteria" group until 2007 and as a single genus from 2008; isolates of other genera in the mixed group are not shown.

'Other' Gram-negative bacteria

The mixed 'other Gram-negative bacteria' collection (2001–07) included 1494 isolates, of which 518 were *Serratia* – counted in Table S2 above and described in the current publication. The next three most numerous genera were *Acinetobacter* (273), *Citrobacter* (198) and *Stenotrophomonas* (182). These were described previously for 2001–2006,[4,5] encompassing most of the period when the group was collected. The 323 remaining isolates belonged to 34 other genera.

Table S3. Antimicrobial data excluded after collection, based on evidence of flawed testing (BSAC surveillance)

Antimicrobial	Year(s)	Organism(s)	Reason
Tigecycline	2002<br>2003	All Gram-negative organisms tested	Optimum testing conditions were unclear in the earliest years and MIC distributions for several organism groups were distorted when compared with later data.
Piperacillin/ tazobactam	2003	*Klebsiella*	The main peak appeared bimodal, with about half of the MICs increased by 2–3 doubling dilutions from the usual values of other years.

Table S4. Numbers of Gram-negative isolates tested by organism subgroup and year (BSAC surveillance)

All isolates were re-identified and tested at the Antimicrobial Resistance and Healthcare-Associated Infections Reference Unit (or its predecessor laboratory) at the now UK Health Security Agency in Colindale, London (previously Public Health Laboratory Service, Health Protection Agency, then Public Health England).[1] Numbers for *Escherichia coli* and *E. cloacae* complex, which were not subdivided by species for analysis, are presented in Table S2.

Year	*Klebsiella*[1]			Proteeae				*Serratia*[2]		*Pseudomonas*	
	K. pneumoniae/variicola	*K. oxytoca*	*K. aerogenes*	*Proteus mirabilis*	*Proteus*, other species	*Morganella morganii*	*Providencia*	*S. marcescens*	*S. liquefaciens*	*P. aeruginosa*	other or no species ID[3]
2001	160	50	22	152	11	34	5	42	8	180	16
2002	184	58	22	155	13	30	5	51	12	187	9
2003	166	69	22	186	13	30	3	66	3	205	17
2004	186	39	23	180	9	21	7	60	9	226	8
2005	174	61	15	175	19	20	8	76	4	216	10
2006	189	48	18	169	10	31	5	81	12	212	5
2007	183	44	32	167	7	28	3	70	3	198	13
2008	147	51	14	157	8	22	2	109	8	190	2
2009	181	42	19	170	8	25	6	115	13	202	10
2010	202	62	31	213	5	32	7	171	15	237	14
2011	200	56	14	205	4	18	2	102	20	209	9
2012	196	55	36	185	5	20	4	137	11	218	13
2013	213	53	44	222	3	18	3	143	9	233	9
2014	205	55	36	210	6	19	2	149	15	223	15
2015	206	57	41	227	5	6	5	142	11	216	11
2016	186	58	29	191	8	8	0	136	15	217	17
2017	174	57	29	208	5	5	0	148	15	208	14
2018	183	52	31	207	2	5	2	139	13	209	12
2019	224	49	12	218	5	5	3	164	9	229	9
Total	3559	1016	490	3597	146	377	72	2101	205	4015	213

[1] 43 *Klebsiella* were not identified to species level (2001–2008) and are not shown.
[2] 40 *Serratia* of other or unrecorded species (2002–2019) (2002–2018) are not shown: 21 unrecorded, 8 *S. odorifera*, 7 *S. rubidaea*, 2 *S. plymuthica* and 1 each of *S. ficaria* and *S. ureilytica*.
[3] 205 *Pseudomonas* belonging to 16 species other than *P. aeruginosa* and 8 not identified to species level.

Table S5. *E. coli:* antibiotics analysed – years included, N of isolates, resistance breakpoints and mode MICs (BSAC surveillance)

Antimicrobial	Years included	N of years	Break-point R > mg/L	*E. coli*	
				N of isolates	Mode MIC mg/L
Amikacin[1]	2014–17	4	8	2068	1
Amoxicillin	2001–19	19	8	7796	≥512‡
Co-amoxiclav[2]	2001–02, 2014–19	8	8	3515	4‡
Cefotaxime[3]	2003–19	17	2	7301	0.06‡
Ceftaroline	2008, 14, 2019	3	0.5	1491	0.12‡
Ceftazidime	2001–19	19	4	7796	0.25‡
Ceftazidime/avibactam[4]	2014, 2017–19	4	8	1976	0.12
Ceftobiprole	2004–10, 2012–19	15	0.25	6531	0.06‡
Ceftolozane/tazobactam[4]	2011–19	9	2	4601	0.12
Cefuroxime	2001–13	13	8	4776	4‡
Ciprofloxacin	2001–19	19	0.5	7796	0.015‡
Colistin[1]	2011–19	9	2	4601	0.5
Ertapenem	2002–07, 2015–19	11	0.5	3956	0.008
Gentamicin[1]	2001–19	19	2	7796	0.5‡
Imipenem	2001–13, 2015–19	18	4	7249	0.12
Imipenem/relebactam[4]	2015–19	5	2	2473	0.12
Meropenem	2006 –08, 2011, 2014–19	10	8	4499	0.015
Piperacillin/tazobactam[4]	2001–19	19	8	7796	2‡
Tigecycline	2004–13	10	0.5	4033	0.25
Tobramycin[1]	2014–19	6	4	3020	0.5‡
Trimethoprim[5]	2015–19	5	4	2473	≥512‡

‡ Most frequent MIC, but distribution was clearly bi- or multi-modal: refer to plot in Appendix.
[1] Epidemiological cut-off (ECOFF) 'breakpoint-in-brackets', intended to identify isolates with acquired resistance mechanisms likely to undermine effectiveness when used as a synergist in combination therapy.
[2] Tested with a fixed 2 mg/L concentration of clavulanate. (The mode MIC for co-amoxiclav tested against 4776 *E. coli* using the now abandoned 2:1 amoxicillin:clavulanate ratio formulation in 2001–2013 was 8 + 4 mg/L.)
[3] Cefotaxime was first tested in 2002, but only at the then-breakpoint concentration of 1 mg/L, so these results are excluded from most analyses; full MICs were determined from 2003 onwards.
[4] Tested with a fixed 4 mg/L concentration of the inhibitor (avibactam, relebactam or tazobactam).
[5] Clinical breakpoint for uncomplicated urinary tract infection only.[6] Trimethoprim is irrelevant to treatment of *E. coli* in blood, but resistance is of interest as *E. coli* bacteraemias commonly originate from the urinary tract and may reflect failure of prior treatment of a UTI.[7]

Table S6. *Klebsiella* spp.: antibiotics analysed – years included, N of isolates, resistance breakpoints and mode MIC (BSAC surveillance)

Antimicrobial	Years included	N of years	Break-point R> mg/L	*K. pneumoniae/variicola*		*K. oxytoca*		*K. aerogenes*	
				N of isolates	Mode MIC mg/L	N of isolates	Mode MIC mg/L	N of isolates	Mode MIC mg/L
Amikacin[1]	2014–17	4	8	771	1‡	227	1	135	1
Co-amoxiclav[2]	2001–02, 2014–19	8	8	1522	2‡	436	1‡	222	≥64
Cefotaxime[3]	2003–19	17	2	3215	0.06‡	908	0.03‡	446	0.12‡
Ceftaroline	2008, 2014, 2019	3	0.5	576	0.12‡	155	0.25‡	62	0.12‡
Ceftazidime	2001–19	19	4	3559	0.25‡	1016	0.12‡	490	0.25‡
Ceftazidime/avibactam[4]	2014, 2017–19	4	8	786	0.25‡	213	0.12	108	0.25
Ceftobiprole	2004–10, 2012–19	15	0.25	2849	0.06‡	783	0.5‡	410	0.06
Ceftolozane/tazobactam[4]	2011–19	9	2	1787	0.25‡	492	0.12	272	0.25
Cefuroxime[5]	2001–13	13	8, x(5)	2381	4‡	688	2‡	312	4‡
Ciprofloxacin[6]	2001–19	19	0.5	3558	0.03‡	1016	0.015	490	0.03
Colistin[1]	2011–19	9	2	1787	0.5	492	0.5	272	0.5
Ertapenem	2002–07 2015–19	11	0.5	2055	0.015	592	0.015	274	0.015‡
Gentamicin[1]	2001–19	19	2	3559	0.5‡	1016	0.5	490	0.5‡
Imipenem	2001–13, 2015–19	18	4	3354	0.5	961	0.25	454	0.5‡
Imipenem/relebactam[4]	2015–19	5	2	973	0.5	273	0.25	142	0.5
Meropenem	2006–08, 2011, 2014–19	10	8	1897	0.03	527	0.03	256	0.03
Piperacillin/tazobactam[4]	2001–02, 2004–19	18	8	3393	4‡	947	2‡	468	4
Tigecycline	2004–13	10	x	1871	0.5	511	0.25	246	0.5‡
Tobramycin[1]	2014–19	6	2	1178	0.5‡	328	0.5	178	0.5

‡ Most frequent MIC, but distribution was clearly bi- or multi-modal: refer to plot in Appendix.
x No breakpoint or epidemiological cut-off (ECOFF) available.
[1, 3, 4] See Table S5.
[2] Tested with fixed 2 mg/L clavulanate. (Mode MICs for co-amoxiclav tested using the now abandoned 2:1 amoxicillin:clavulanate ratio formulation in 2001–2013 were 2 + 1 mg/L for *K. pneumoniae/variic*ola (N=2381) and *K. oxytoca* (N=688) and 32 + 16 mg/L for *K. aerogenes* (N=312).
[5] Cefuroxime breakpoint does not apply to *K. aerogenes*, which is inherently resistant.
[6] One ciprofloxacin result for *K. pneumoniae/ variicola* excluded as uninterpretable (recorded as ‘0.256 mg/L’).

Table S7. *E. cloacae* complex: antibiotics analysed – years included, N of isolates, resistance breakpoints and mode MIC (BSAC surveillance)

Antimicrobial	Years included	N of years	Break-point R> mg/L	*E. cloacae* complex	
				N of isolates	Mode MIC mg/L
Amikacin[1]	2014–17	4	8	668	1
Cefotaxime[3]	2003–19	17	2	2756	0.25‡
Ceftaroline	2008, 2014, 2019	3	0.5	463	0.25‡
Ceftazidime	2001–19	19	4	3064	0.25‡
Ceftazidime/avibactam[4]	2014, 2017–19	4	8	669	0.25
Ceftobiprole	2004–10, 2012–19	15	0.25	2425	0.06‡
Ceftolozane/tazobactam[4]	2011–19	9	2	1487	0.25‡
Ciprofloxacin	2001–19	19	0.5	3064	0.015‡
Colistin[1]	2011–19	9	2	1487	0.5‡
Ertapenem	2002–07 2015–19	11	0.5	1824	0.015‡
Gentamicin[1]	2001–2019	19	2	3064	0.5‡
Imipenem	2001–13, 2015–19	18	4	2892	0.5
Imipenem/relebactam[4]	2015–19	5	2	836	0.25
Meropenem	2006 –08, 2011, 2014–19	10	8	1590	0.03
Piperacillin/tazobactam[4]	2001–19	19	8	3064	2‡
Tigecycline	2004–13	10	x	1575	0.5
Tobramycin[1]	2014–19	6	2	1008	0.5‡

‡ Most frequent MIC, but distribution was clearly bi- or multi-modal: refer to plot in Appendix.
[1, 3, 4] See Table S5; ([2] Not relevant to *Enterobacter*.)

Table S8. Proteeae: antibiotics analysed – years included, N of isolates, resistance breakpoints and mode MIC (BSAC surveillance)

Antimicrobial	Years included	N of years	Break-point R > mg/L	*Proteus mirabilis*		*M. morganii*	
				N of isolates	Mode MIC mg/L	N of isolates	Mode MIC mg/L
Amikacin[1(a)]	2014–17	4	16, 8[(a)]	836	2	38	2
Amoxicillin	2001–19	19	8	3597	0.5‡	(x)	(x)
Co-amoxiclav[2]	2001–02, 2014–19	8	8	1568	0.5‡	(x)	(x)
Cefotaxime[3]	2003–19	17	2	3290	≤0.008‡	313	≤0.015‡
Ceftaroline	2008, 2014, 2019	3	0.5	585	0.06	46	0.03
Ceftazidime	2001–19	19	4	3597	0.03‡	377	0.03‡
Ceftazidime/avibactam[4]	2014, 2017–19	4	8	843	0.03	34	0.015
Ceftobiprole	2004–10, 2012–19	15	0.25	2899	0.03‡	265	0.03
Ceftolozane/tazobactam[4]	2011–19	9	2	1873	0.12	104	0.12
Cefuroxime	2001–13	13	8	2336	1‡	(x)	(x)
Ciprofloxacin	2001–19	19	0.5	3597	0.03‡	377	0.015‡
Ertapenem	2002–07 2015–19	11	0.5	2083	0.008	189	0.008
Gentamicin[1(a)]	2001–19	19	4, 2[(a)]	3597	0.5	377	0.5‡
Imipenem	2001–13, 2015–19	18	4	3387	2‡	358	2
Imipenem/relebactam[4]	2015–19	5	x	1051	2	29	2
Meropenem	2006 –08, 2011, 2014–19	10	8	1959	0.06	147	0.06
Piperacillin/tazobactam[4]	2001–19	19	8	3597	0.25	377	0.12
Tigecycline	2004–13	10	x	1843	4	235	2
Tobramycin[1]	2014–19	6	4	1261	1‡	48	1

‡ Most frequent MIC, but distribution was clearly bi- or multi-modal: refer to plot in Appendix.

x No breakpoint or epidemiological cut-off (ECOFF) available. (x) antimicrobial not relevant to *Morganella*.

[1, 3, 4] See Table S5.

[2] Tested with fixed 2 mg/L clavulanate. (The mode MIC for co-amoxiclav tested using the now abandoned 2:1 amoxicillin:clavulanate ratio in 2001–2013 was ≤(1 + 0.5) mg/L for *P. mirabilis* (N=2336)).

[(a)] ECOFFs for *M. morganii*, where different from *P. mirabilis*: amikacin R >8, gentamicin R >2 mg/L.

Table S9. *Serratia*: antibiotics analysed – years included, N of isolates, resistance breakpoints and mode MIC (BSAC surveillance)

Antimicrobial	Years included	N of years	Break-point R > mg/L	*Serratia* spp.	
				N of isolates	Mode MIC mg/L
Amikacin[1(a)]	2014–17	4	8, x[(a)]	638	2
Cefotaxime[3]	2003–19	17	2	2231	0.25‡
Ceftaroline	2008, 2014, 2019	3	0.5	457	1‡
Ceftazidime	2001–19	19	4	2346	0.25‡
Ceftazidime/avibactam[4]	2014, 2017–19	4	8	657	0.25
Ceftobiprole	2004–10, 2012–19	15	0.25	2037	0.12
Ceftolozane/tazobactam[4]	2011–19	9	2	1390	0.5
Ciprofloxacin	2001–19	19	0.5	2346	0.12‡
Ertapenem	2002–07 2015–19	11	0.5	1267	0.03
Gentamicin[1]	2001–19	19	2, x[(a)]	2346	0.5‡
Imipenem	2001–13, 2015–19	18	4	2181	0.5
Imipenem/relebactam[4]	2015–19	5	2	799	0.25
Meropenem	2006 –08, 2011, 2014–19	10	8	1381	0.03
Piperacillin/tazobactam[4]	2001–19	19	8	2346	2‡
Tigecycline	2004–13	10	x	1197	1
Tobramycin[1(a)]	2014–19	6	8, x[(a)]	964	2

‡ Most frequent MIC, but distribution was clearly bi- or multi-modal: refer to plot in Appendix.
[1, 3, 4] See footnotes to Table S5; ([2] Not relevant to *Serratia*.)
[(a)] Specific ECOFFs have not been established for *Serratia* species other than *marcescens*.

The mode MICs shown are for *Serratia* as a whole and were identical for *S. marcescens*, which comprised 91% of isolates. The modes for *S. liquefaciens* were within ± 1 dilution of those for *S. marcescens* for all antimicrobials except ciprofloxacin (0.015 mg/L) and tobramycin (0.25 mg/L), where the modes for *S. liquefaciens* were 3 doubling dilutions lower – see MIC distributions.

Table S10. *Pseudomonas*: antibiotics analysed – years included, N of isolates, resistance breakpoints and mode MIC (BSAC surveillance)

Antimicrobial	Years included	N of years	Break-point R > mg/L	*P. aeruginosa**	
				N of isolates	Mode MIC mg/L
Amikacin[1]	2014–2017	4	16	864	2
Ceftazidime	2001–2019	19	8	4015	2[‡]
Ceftazidime/avibactam	2014, 2017–2019	4	8	869	2
Ceftobiprole	2004–2010, 2012–2019	15	-	3234	2
Ceftolozane/tazobactam[4]	2011–2019	9	4	1962	0.5
Ciprofloxacin	2001–2019	19	0.5	4015	0.12[‡]
Colistin	2011–2019	9	4	1962	1
Gentamicin	2001–2019	19	8	4015	1[‡]
Imipenem	2001–2013, 2015–2019	18	4	3792	1
Imipenem/relebactam[4]	2015–2019	5	2	1079	0.25
Meropenem	2006–2008, 2011, 2014–2019	10	8	2111	0.25
Piperacillin/tazobactam[4]	2001–2019	19	16	4015	4
Tobramycin	2014–2019	6	2	1302	0.25[‡]

* Excludes 205 isolates of 16 other *Pseudomonas* species and 8 identified only to genus level. Mode MICs of amikacin, gentamicin and tobramycin, at ≤0.12 mg/L, were distinctly lower for these isolates (as a group than) than for *P. aeruginosa*. Their mode MICs for other agents (except meropenem, 2 mg/L) were within ±1 dilution of those for *P. aeruginosa*.

[‡] Most frequent MIC, but distribution was clearly bi- or multi-modal: refer to plot in Appendix.

[1, 4] See footnotes to Table S5; ([2,3] Not relevant to *Pseudomonas*).

BSAC bacteraemia resistance surveillance – patient characteristics

Sex

In general, as commonly noted,[8] and except for *E. coli* (48%) there was a preponderance of male patients, ranging from 60% (*Klebsiella, Enterobacter*) to 64% (*Serratia*). There were also clear differences between organism subgroups.

Table S11. Proportion (%) of male patients by organism species

Gram-negative		
Organism group	N[1]	Male, %
E. coli	7781	48.0
K. pneumoniae/variicola	3553	58.4
K. oxytoca	1013	62.1
K. aerogenes	488	69.5
Klebsiella spp. ††	43	53
E. cloacae complex	3056	60.3
Proteus mirabilis	3591	63.0
Proteus other species	146	68.5
Morganella morganii	377	64.7
Providencia spp.†	72	74
S. marcescens	2099	65.1
S. liquefaciens	202	56.4
Serratia: other/spp.††	43	60
P. aeruginosa	4005	61.5
Pseudomonas: other/spp.	213	48.8

[1] Number of isolates with sex data for the source patient. (Missing: 52/26,734 = 0.2%)
†† Caution: ≤50 isolates; imprecise estimates.

Age

All organism groups had infants aged under one year old as a distinct subgroup of source patients, largest for *Enterobacter* at 6%. Subgroups with similarly high proportions of infants were *K. oxytoca* and *K. aerogenes* at 5%. The highest proportions of patients aged 80 and over were seen in bacteraemias caused by Proteeae (38%) and *E. coli* (33%)

Table S12. Patient age: summary measures by organism group

Organism group	N[1]	Quartiles, years Q1	Q2	Q3	Isolates in age group shown, % <1 year	≥65 years	≥80 years
E. coli	7767	60	73	82	1.9	67.8	33.1
Klebsiella	5085	56	70	79	2.8	60.9	23.3
Enterobacter	3052	45	63	75	6.2	47.8	16.3
Proteeae	4171	64	76	83	0.4	73.8	37.9
Serratia	2336	55	68	78	2.6	58.8	19.9
Pseudomonas	4194	55	69	79	2.1	59.7	23.1

[1] Number of isolates with age data for the source patient. (Missing: 129/26734 = 0.5%)

[2] Patients aged ≥80 years are an older subset of those who are ≥65 years old

Figure S1. Patient age: histograms and trends by organism group

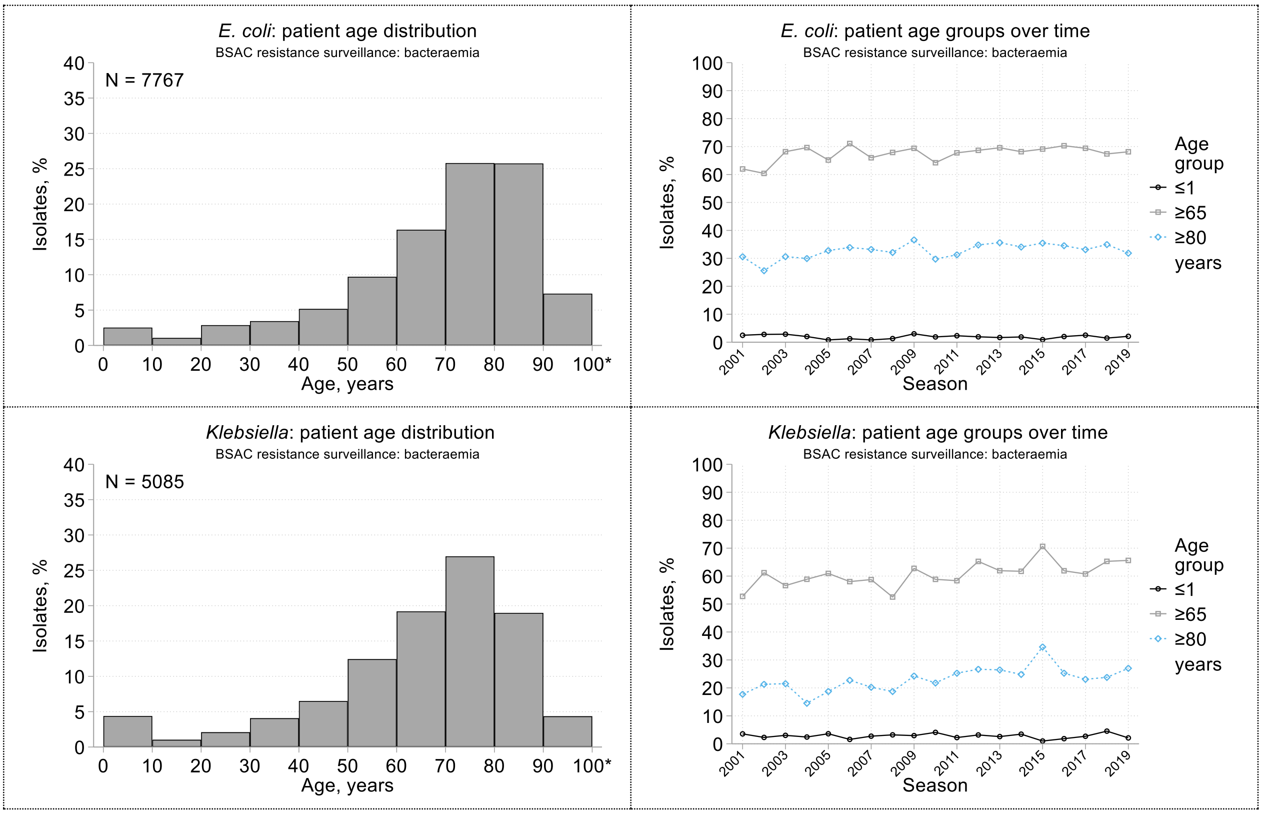

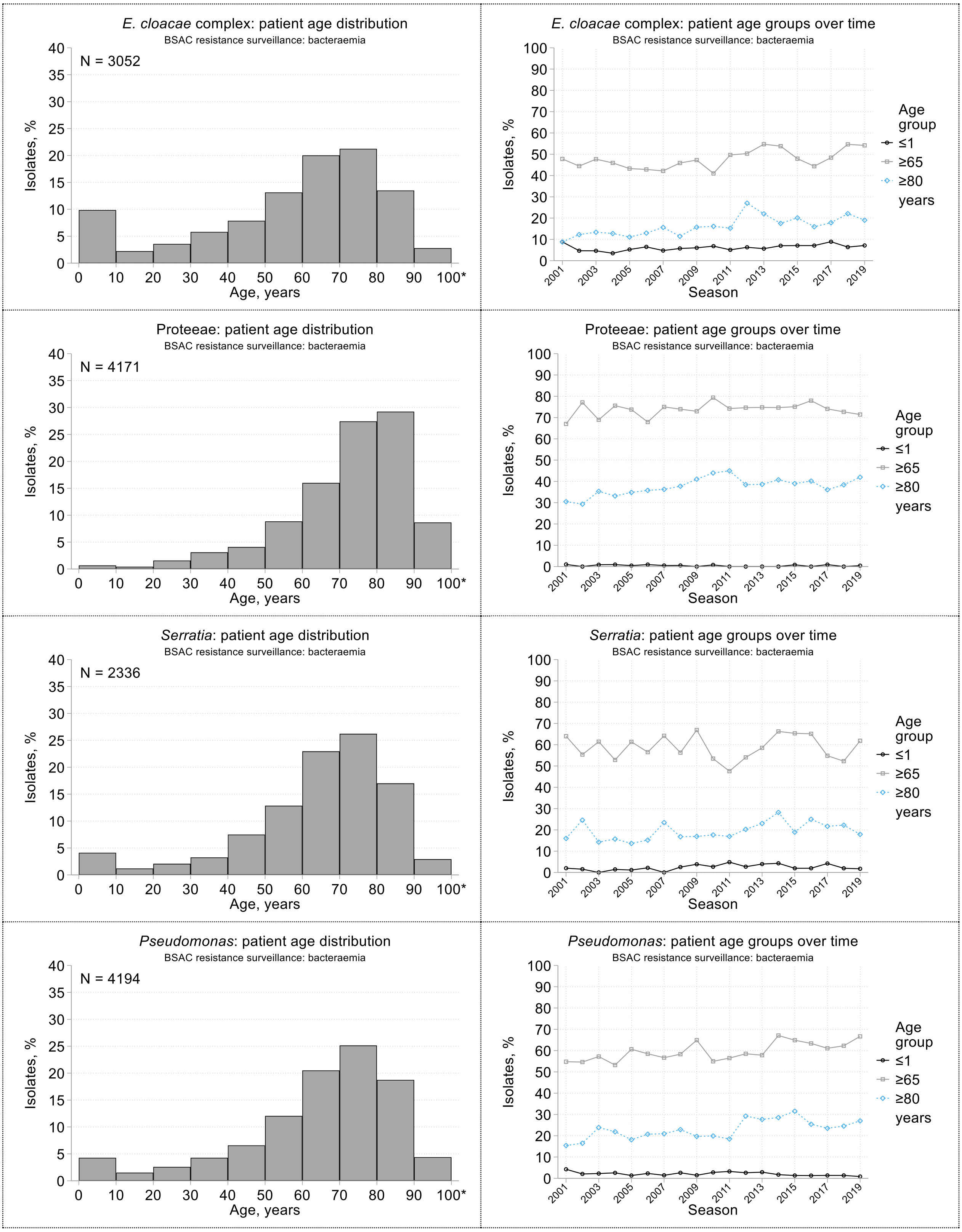

* The final bar includes all patients aged ≥90 years.

Care setting – hospitalised >48 hours at time of sample versus any other care setting (community, outpatients, hospitalised ≤48 hours)

The proportion of isolates from patients in hospital for >48 hours fell substantially over time for all groups. The proportion from community and outpatients increased to a peak around 2012 before dropping back, and the proportion in hospital for less than ≤48 hours increased substantially in the later part of the surveillance. Care setting data was seldom missing from 2003 onwards (0.5%), but more often (9.2%) in 2001–02, before improvements to data collection forms.

Figure S2. Trends in care setting by organism group

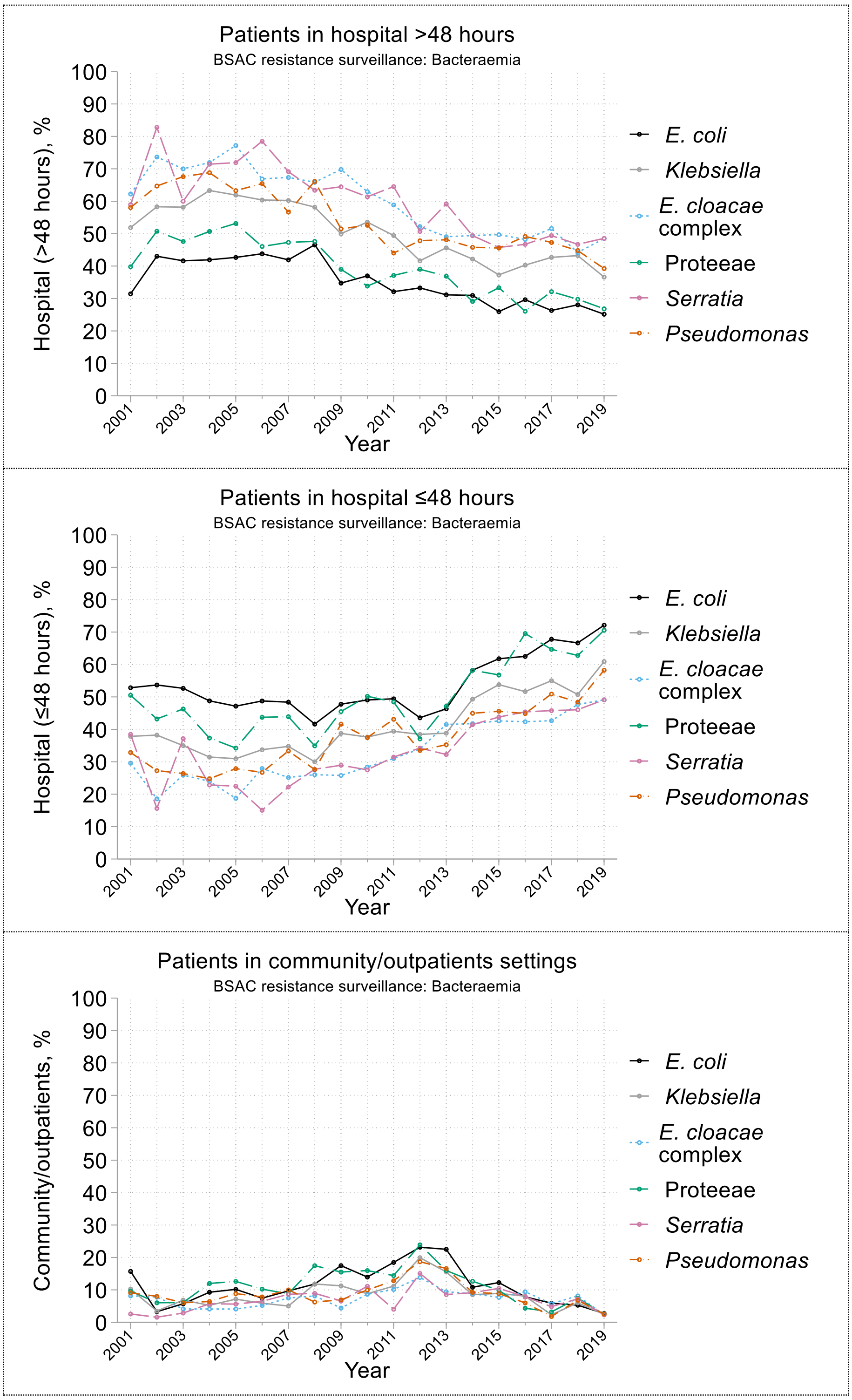

Care setting: intensive/critical care speciality (ICU)

Hospital speciality data from 2001–02 were discrepant and are excluded: data collection forms were improved from 2003 onwards after noting issues with recording in the 2001 data. The lowest proportion of ICU care was for patients with bacteraemias caused by *E. coli* (4%) and Proteeae (5%).

Table S13. Proportion of isolates from ICU patients, by organism group

	Gram-negative	
Organism group	N[1]	ICU, %
E. coli	7008	4.0
Klebsiella	4446	11.1
Enterobacter	2681	14.3
Proteeae	3645	4.7
Serratia	2153	13.7
Pseudomonas	3721	12.2

[1] Number of isolates with speciality data, after excluding 2001–02. (Missing data: 846/24,500 = 3.5%; varied slightly between organisms from 2.7% for *Enterobacter* to 4.0% for *E.coli*.)

Caveat: Tabulated 'overall' figures do not represent any particular year, if the ICU proportion changed over time (see Figure S3).

Figure S3. Trends in proportion of isolates from ICU patients by organism group

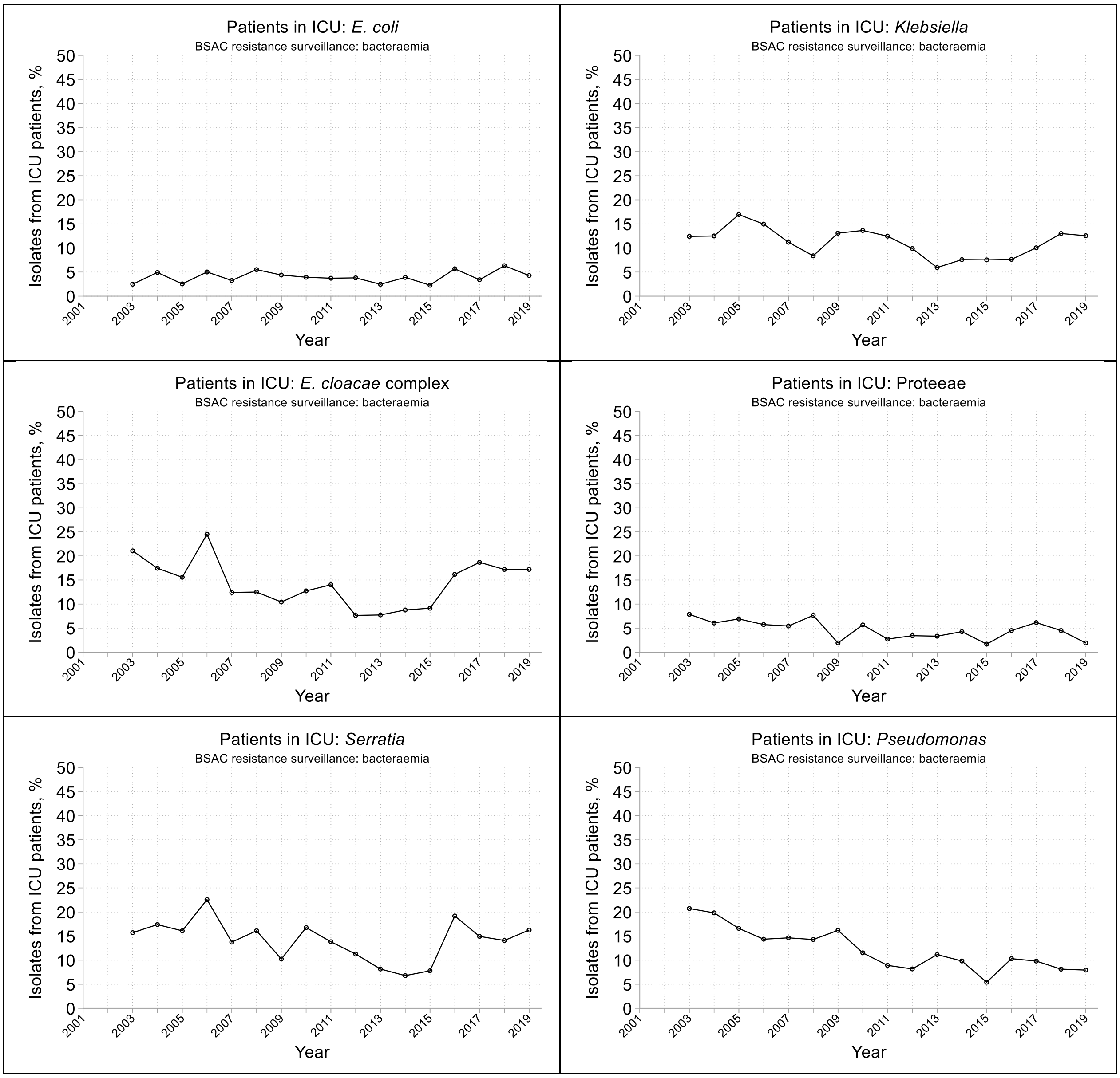

Source of bacteraemia (2001–13)

From 2001–13, the foci of infection, and putative sources of bacteraemia, were recorded as the genitourinary tract, lines, respiratory tract, gastrointestinal tract or skin and soft tissue, endocarditis, surgical site, cerebrospinal fluid, or other, but the data quality was poor, with 2% listed as 'other' and 38% missing. In 2014–15, free text entries were accepted but were too disparate for meaningful analysis, and data collection was abandoned from 2016 onwards.

Table S14. Source of infection: top three or ≥10% of isolates, by organism

Organism group	N[1]	Major sources of bacteraemia
E. coli	3101	genitourinary (63%), gastrointestinal (20%), respiratory (6%)
Klebsiella	2099	genitourinary (35%), gastrointestinal (26%), lines (19%), respiratory (12%)
Enterobacter	1204	lines (32%), genitourinary (23%), gastrointestinal (22%), respiratory (10%)
Proteeae	1841	genitourinary (66%), gastrointestinal (9%), lines (8%)
Serratia	776	lines (27%), genitourinary (21%), gastrointestinal (19%), respiratory (15%)
Pseudomonas	1718	genitourinary (29%), lines (25%), respiratory (19%), gastrointestinal (12%)

[1] Number of isolates 2001–13 with data for presumed focus of infection.
Caution: 38% missing data; recording may have been of poor quality.

UKHSA voluntary bacteraemia surveillance – routine data, England only

Table S15. Number of reported Gram-negative bacteraemias, by pathogen group (UKHSA surveillance, routine data)

Year	*E. coli*	*Klebsiella*[1] spp.	*Entero-bacter*[1] spp.	Proteeae	*Serratia* spp.	*Pseudo-monas* spp.
2001	11423	3210	1470	1847	556	2289
2002	12494	3609	1648	2030	647	2426
2003	14784	4189	2009	2315	819	3005
2004	15453	4669	1986	2254	896	2906
2005	16606	4848	2002	2203	917	3109
2006	17895	5293	2114	2249	1056	3501
2007	19691	5756	2155	2519	1000	3743
2008	21542	5815	1856	2766	904	3794
2009	23131	5969	1760	2829	872	3747
2010	24524	5931	1555	2721	828	3675
2011	26658	6310	1561	2837	777	3539
2012	27257	6401	1420	2798	735	3624
2013	27882	6300	1473	2788	728	3470
2014	28617	6760	1479	2925	796	3587
2015	31331	7373	1547	3193	844	3906
2016	34771	8508	1762	3797	932	4090
2017	36379	9168	1972	4006	1017	4532
2018	37718	9539	2060	4081	1028	4379
2019	39907	10391	2212	4364	1195	4615
2020	35250	10234	2348	4295	1219	4518
2021	35330	11033	2668	4396	1459	4959
2022	35737	11032	2575	4702	1300	4778
2023	38772	12186	2578	4842	1462	4888
2024	40691	12544	2462	4902	1539	4943

[1] After reclassifying *Enterobacter aerogenes* into *Klebsiella*.
Counts of bacteraemia episodes for 2020–2024 are presented here and in Figure S4, showing the continuing importance of the organism groups included in the BSAC surveillance, but we did not analyse them for antimicrobial resistance as we could not compare with BSAC surveillance after 2019.

Figure S4. Number of reported Gram-negative bacteraemias with organisms included in BSAC surveillance (UKHSA surveillance, routine data)

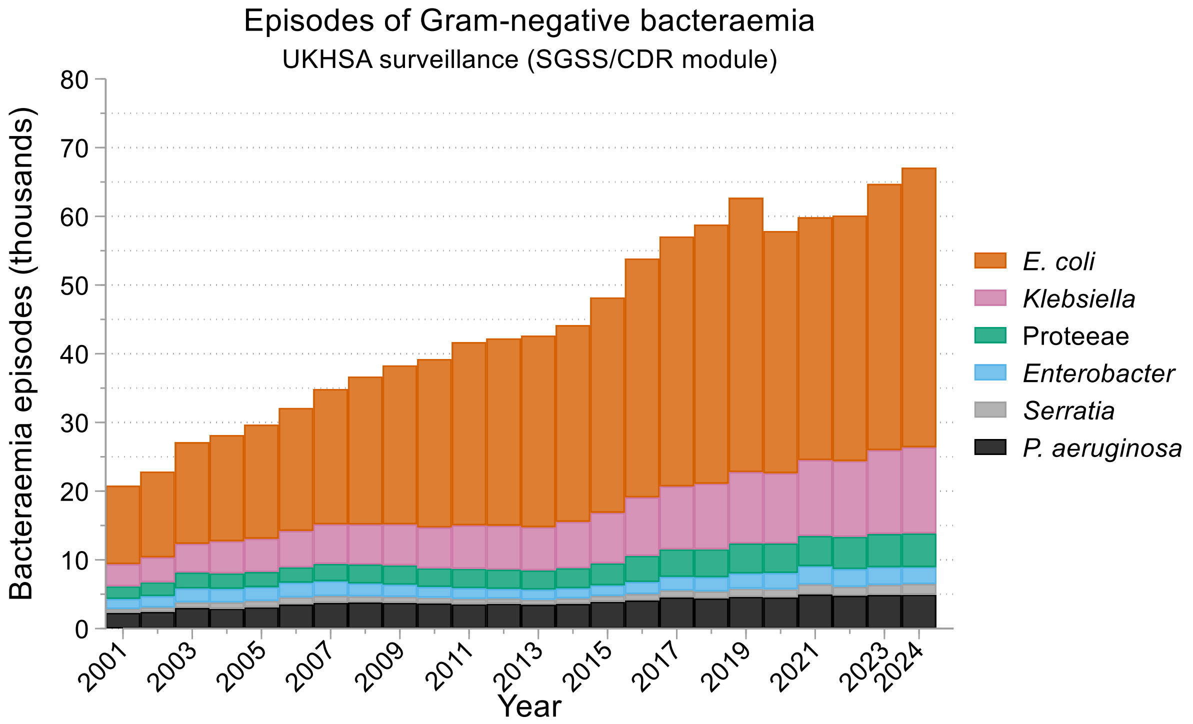

Figure S5. Trends in percentage of bacteraemia episodes reported to UKHSA with antimicrobial susceptibility test results, by organism group (UKHSA surveillance)

KEY to antimicrobials: resistance data present for ...

AMP: ampicillin
AMX: amoxicillin
AMC: co-amoxiclav
3G-ceph: cefotaxime +/- ceftazidime +/- ceftriaxone
CIP: ciprofloxacin
GEN: gentamicin
IPM: imipenem
MEM: meropenem
TZP: piperacillin/tazobactam

+/- means and/or

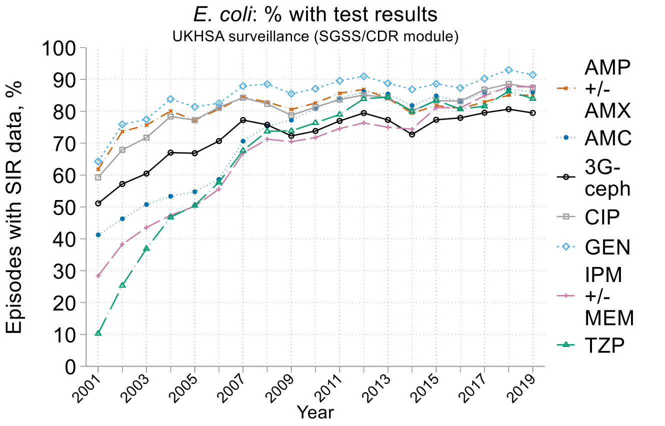

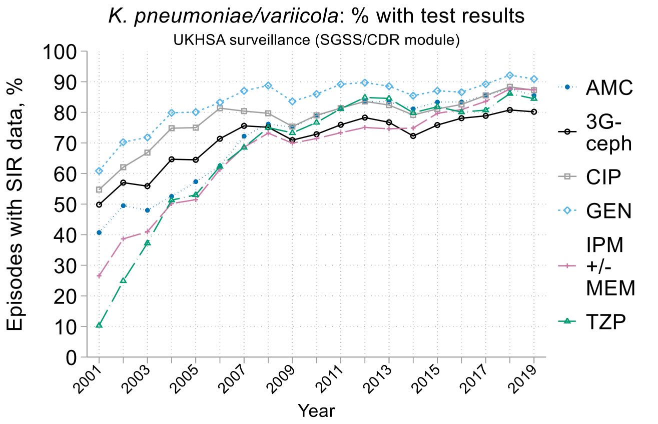

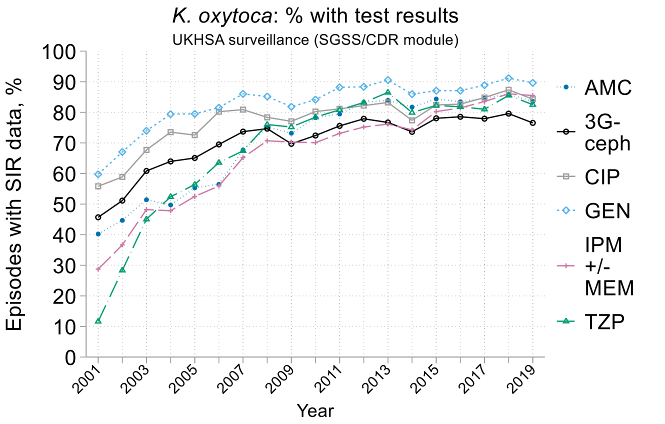

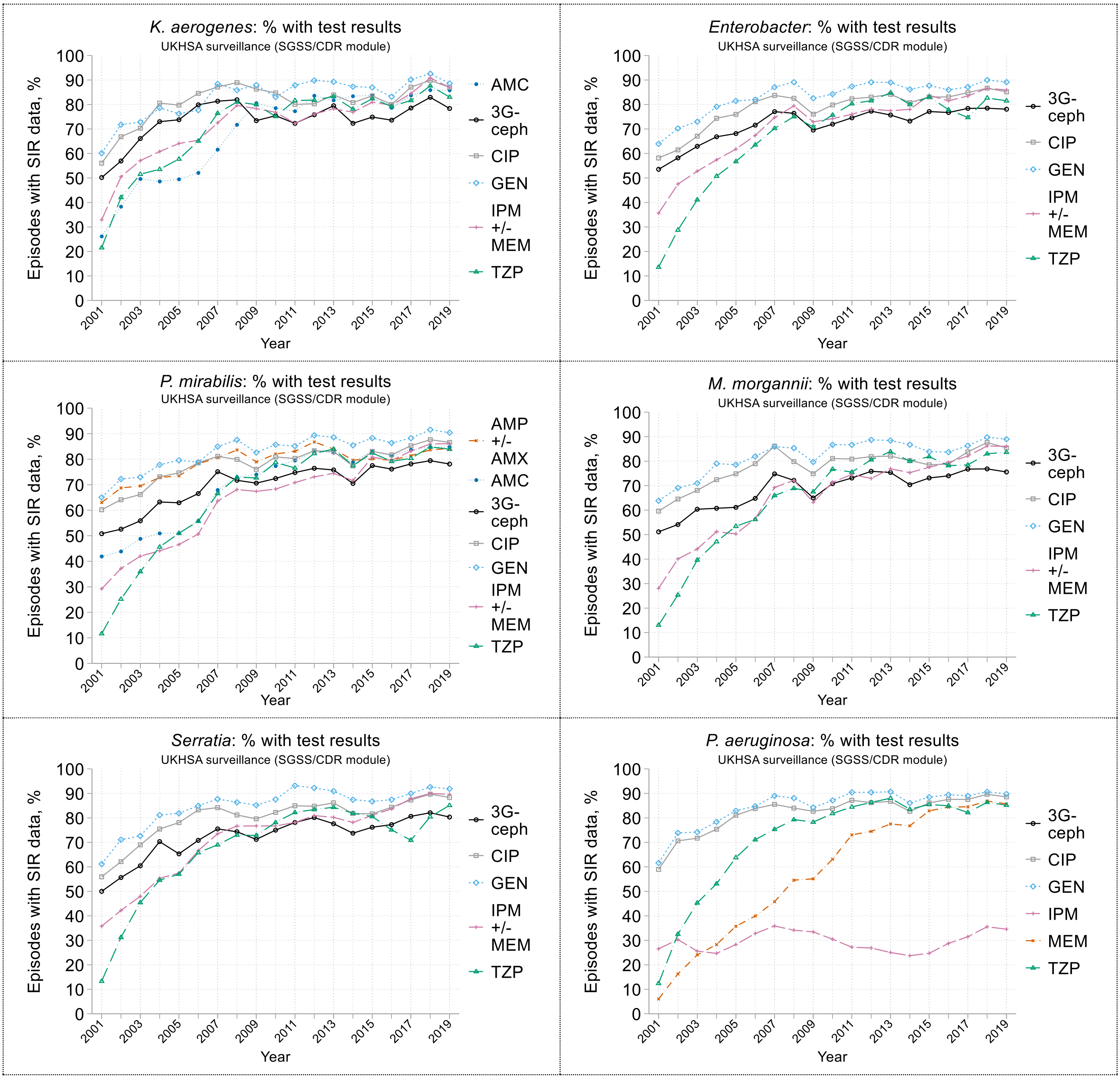

K. aerogenes: % with test results
UKHSA surveillance (SGSS/CDR module)
Episodes with SIR data, %
Year
AMC
3G-ceph
CIP
GEN
IPM +/- MEM
TZP
Enterobacter: % with test results
UKHSA surveillance (SGSS/CDR module)
Episodes with SIR data, %
Year
3G-ceph
CIP
GEN
IPM +/- MEM
TZP
P. mirabilis: % with test results
UKHSA surveillance (SGSS/CDR module)
Episodes with SIR data, %
Year
AMP +/- AMX
AMC
3G-ceph
CIP
GEN
IPM +/- MEM
TZP
M. morgannii: % with test results
UKHSA surveillance (SGSS/CDR module)
Episodes with SIR data, %
Year
3G-ceph
CIP
GEN
IPM +/- MEM
TZP
Serratia: % with test results
UKHSA surveillance (SGSS/CDR module)
Episodes with SIR data, %
Year
3G-ceph
CIP
GEN
IPM +/- MEM
TZP
P. aeruginosa: % with test results
UKHSA surveillance (SGSS/CDR module)
Episodes with SIR data, %
Year
3G-ceph
CIP
GEN
IPM
MEM
TZP

References

1. Allen M, Reynolds R, Mushtaq S *et al*. The British Society for Antimicrobial Chemotherapy Resistance Surveillance Project: methods and limitations. *J Antimicrob Chemother* 2025; **80** (Suppl 4): iv7–iv21.

2. Reacher MH, Shah A, Livermore DM *et al.* Bacteraemia and antibiotic resistance of its pathogens reported in England and Wales between 1990 and 1998: trend analysis. *BMJ* 2000; **320**: 213–6.

3. Wilson J, Elgohari S, Livermore DM *et al.* Trends among pathogens reported as causing bacteraemia in England, 2004-2008. *Clin Microbiol Infect* 2011; **17**: 451–8.

4. Livermore DM, Hope R, Brick G *et al.* Non-susceptibility trends among Enterobacteriaceae from bacteraemias in the UK and Ireland, 2001-06. *J Antimicrob Chemother* 2008; **62 Suppl 2**: ii41-54.

5. Livermore DM, Hope R, Brick G *et al*. Non-susceptibility trends among *Pseudomonas aeruginosa* and other non-fermentative Gram-negative bacteria from bacteraemias in the UK and Ireland, 2001-06. *J Antimicrob Chemother* 2008; **62 Suppl 2**: ii55-63.

6. Anon. EUCAST: Clinical breakpoints and dosing of antibiotics. Available at: https://www.eucast.org/clinical_breakpoints/

7. Abernethy J, Guy R, Sheridan EA *et al.* Epidemiology of *Escherichia coli* bacteraemia in England: results of an enhanced sentinel surveillance programme. *J Hosp Infect* 2017; **95**: 365–75.

8. Christaki E, Giamarellos-Bourboulis EJ. The complex pathogenesis of bacteremia. *Virulence* 2014; **5**: 57–65.

APPENDIX – MIC distributions

BSAC Gram-negative bacteraemia resistance surveillance

These graphs are presented as thumbnails for reasons of space. Please zoom in to read.
The red vertical lines show EUCAST v12.0 (2022) breakpoint(s)[6] or, if used for analysis in the absence of breakpoints, ECOFFs – see Tables S4–S10. Where two lines are shown, they indicate the susceptible (S ≤) and resistant (R >) breakpoints; MICs between these bounds are designated I "susceptible, increased exposure". More commonly, there is a single line because the S and R breakpoints are coincident and there is no I category. Occasionally, noted below, there is no S category, and the single line demarcates R from I.

Some distributions were affected by excessive censoring due to testing of restricted concentration ranges, usually in earlier years. These years' data are omitted, as noted in the affected plots, to show the true range more accurately.

Collection years and number of isolates are noted within each plot. The MIC axes all span ≤0.001 to ≥1024 mg/L, with labelled values showing the range of MICs actually observed in those years.

Combinations lacking S category
Enterobacterales: cefuroxime (all); imipenem (Proteeae)
Pseudomonas: ceftazidime, ciprofloxacin, imipenem, piperacillin/tazobactam

E. coli

E. coli

Amikacin

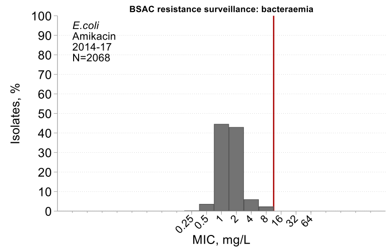

Amoxicillin

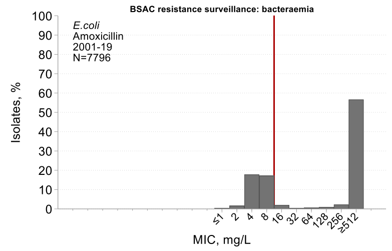

Co-amoxiclav (2:1 ratio)

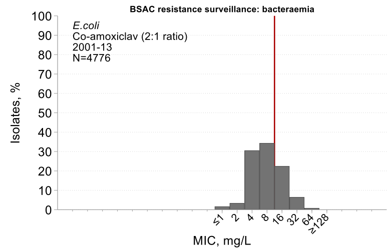

Co-amoxiclav (2 mg/L clavulanate)

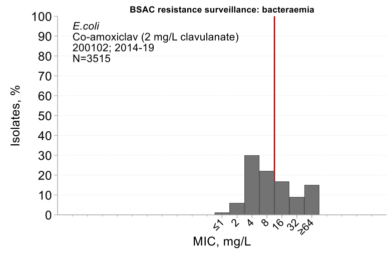

Cefotaxime

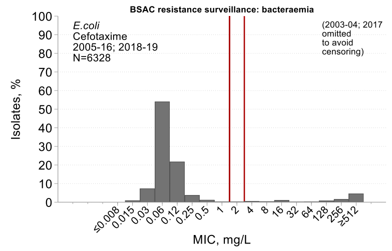

Ceftaroline

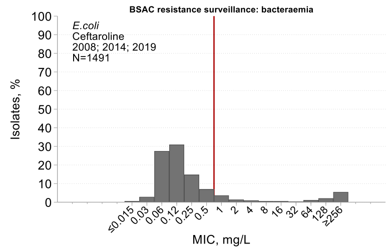

Ceftazidime

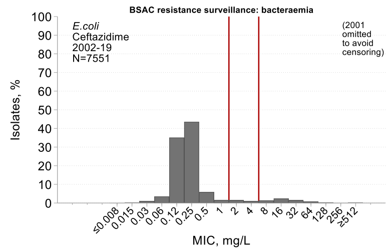

Ceftazidime/avibactam

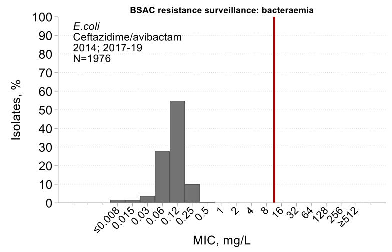

Ceftobiprole

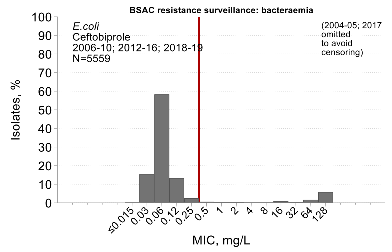

E. coli

Ceftolozane/tazobactam

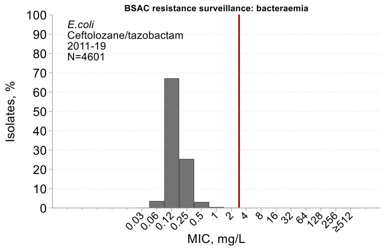

Cefuroxime

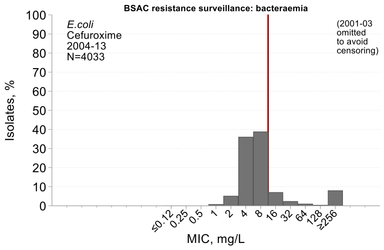

Ciprofloxacin

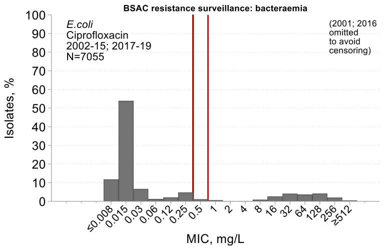

Colistin

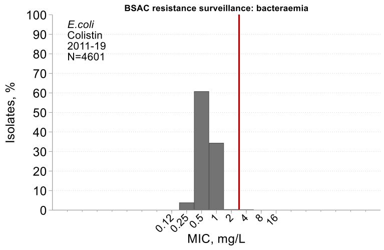

Ertapenem

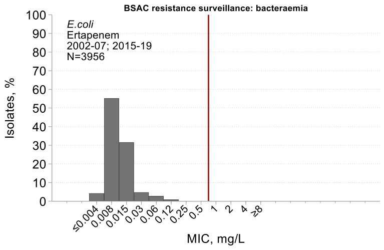

Gentamicin

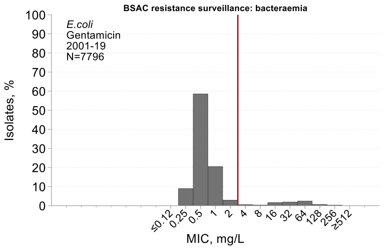

Imipenem

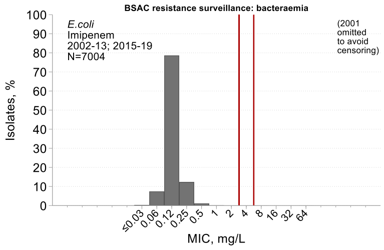

Imipenem/relebactam

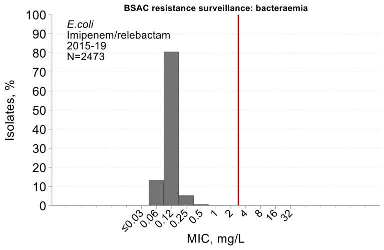

Meropenem

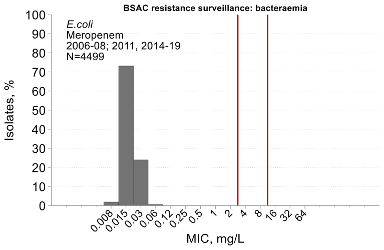

Piperacillin/tazobactam

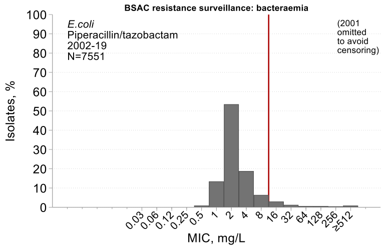

Tigecycline

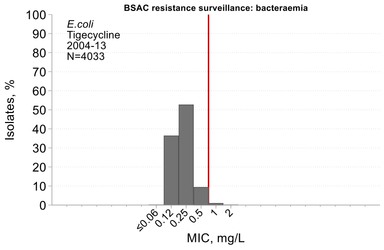

Tobramycin

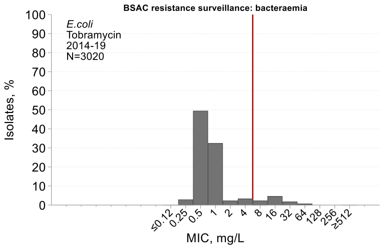

Trimethoprim

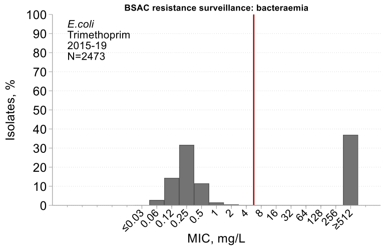

Klebsiella spp.

K. pneumoniae/variicola	*Klebsiella* *K. oxytoca*	*K. aerogenes*
**Amikacin** 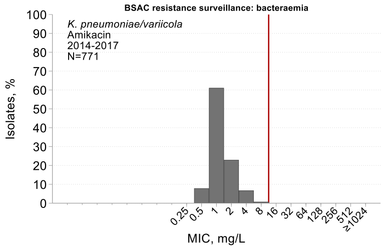	**Amikacin** 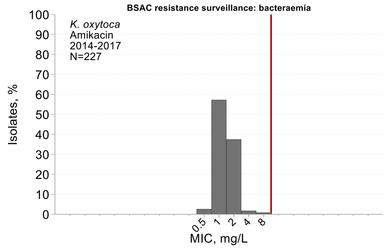	**Amikacin** 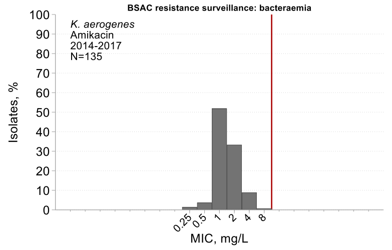
**Co-amoxiclav (2:1 ratio)** 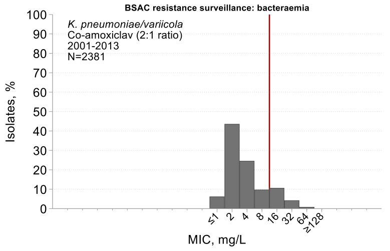	**Co-amoxiclav (2:1 ratio)** 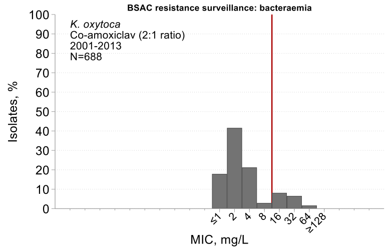	**Co-amoxiclav (2:1 ratio)** 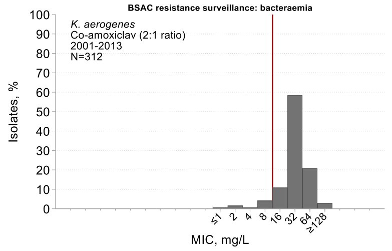
**Co-amoxiclav (2 mg/L clavulanate)** 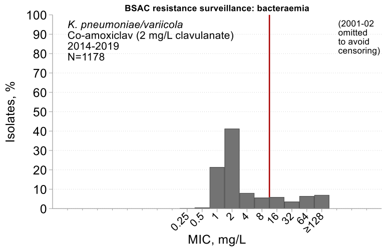	**Co-amoxiclav (2 mg/L clavulanate)** 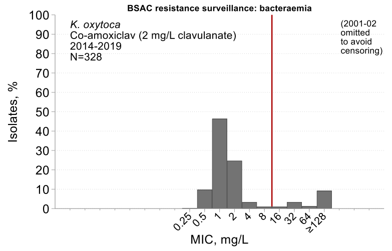	**Co-amoxiclav (2 mg/L clavulanate)** 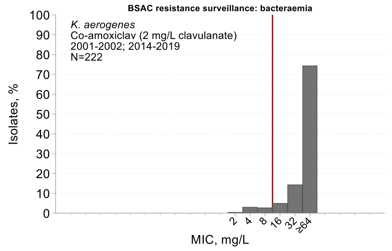
**Cefotaxime** 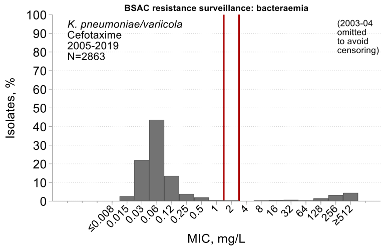	**Cefotaxime** 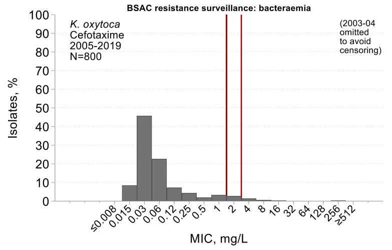	**Cefotaxime** 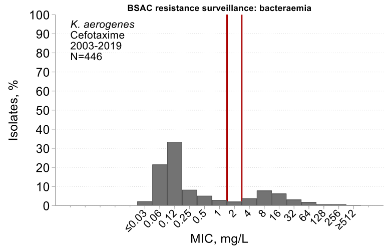
**Ceftaroline** 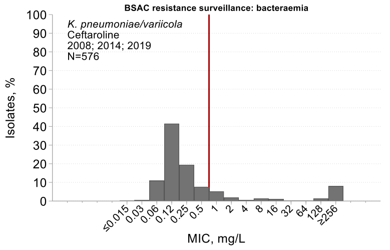	**Ceftaroline** 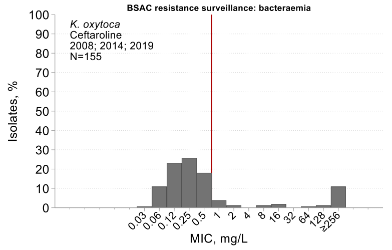	**Ceftaroline** 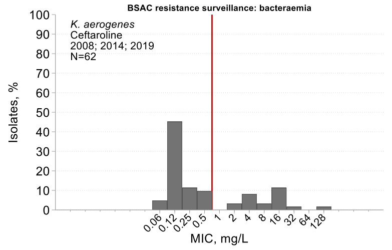
**Ceftazidime** 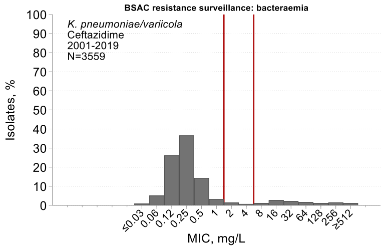	**Ceftazidime** 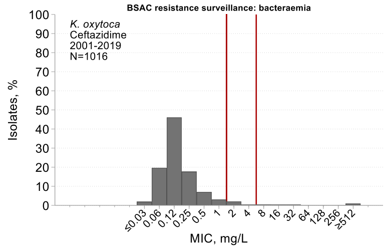	**Ceftazidime** 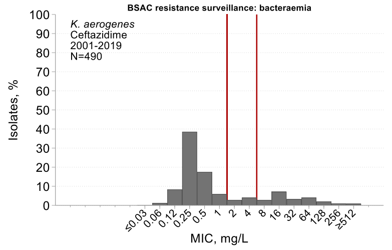

Klebsiella

K. pneumoniae/variicola	*K. oxytoca*	*K. aerogenes*

Ceftazidime/avibactam

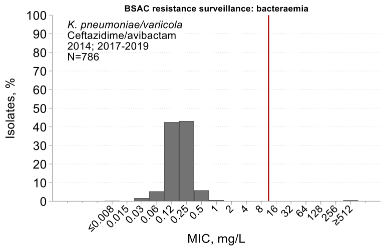

Ceftazidime/avibactam

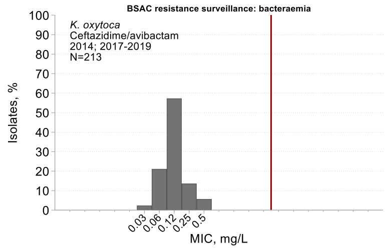

Ceftazidime/avibactam

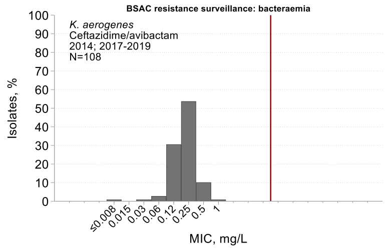

Ceftobiprole

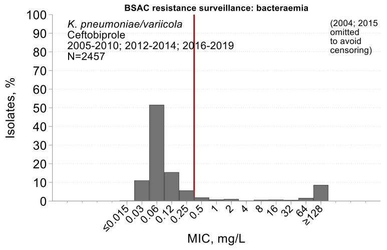

Ceftobiprole

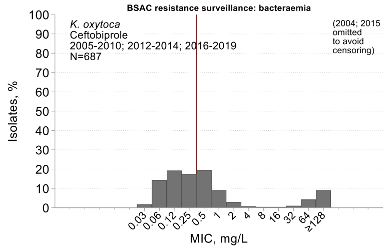

Ceftobiprole

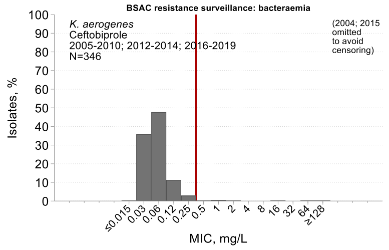

Ceftolozane/tazobactam

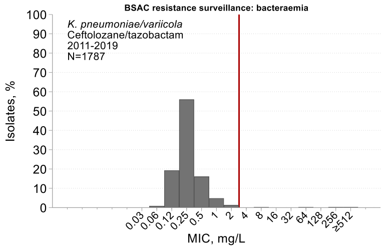

Ceftolozane/tazobactam

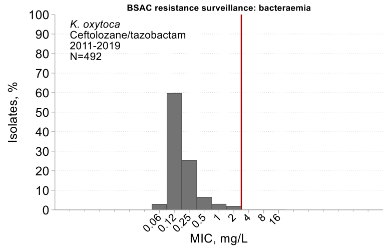

Ceftolozane/tazobactam

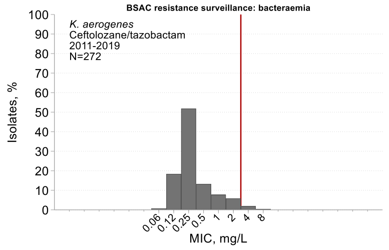

Cefuroxime

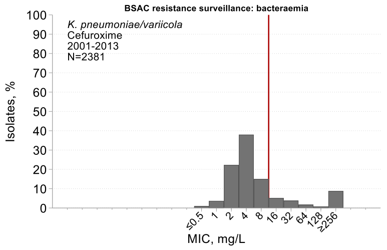

Cefuroxime

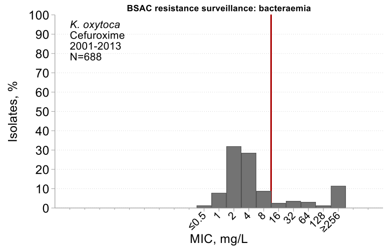

Cefuroxime

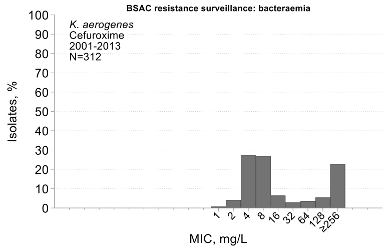

Ciprofloxacin

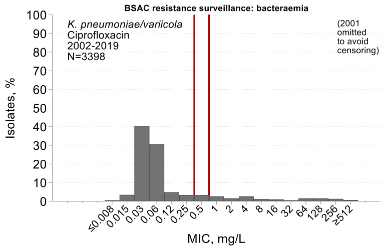

Ciprofloxacin

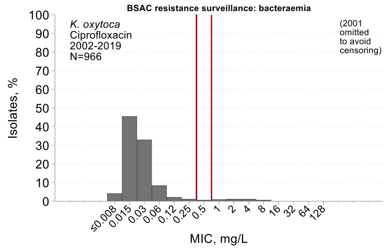

Ciprofloxacin

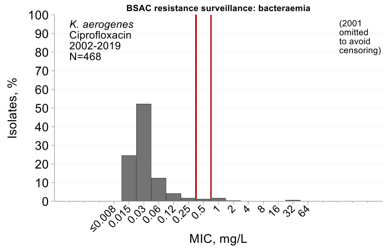

Colistin

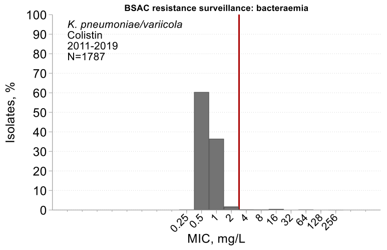

Colistin

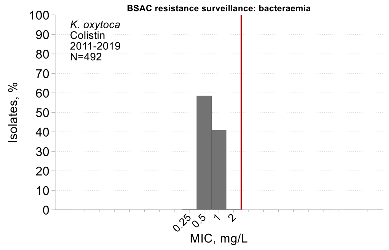

Colistin

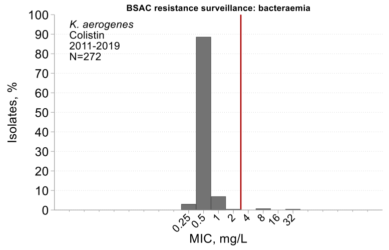

Klebsiella

K. pneumoniae/variicola	*K. oxytoca*	*K. aerogenes*

Ertapenem

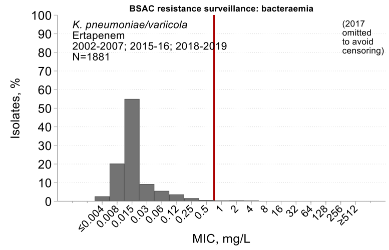

Ertapenem

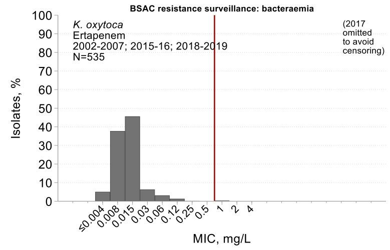

Ertapenem

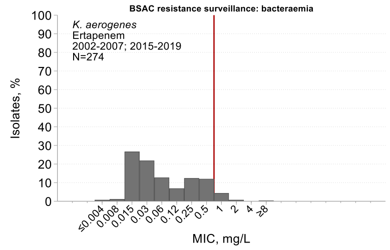

Gentamicin

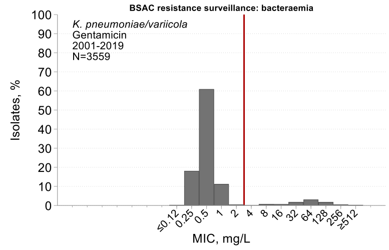

Gentamicin

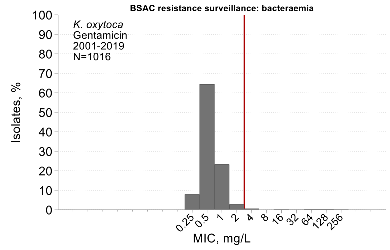

Gentamicin

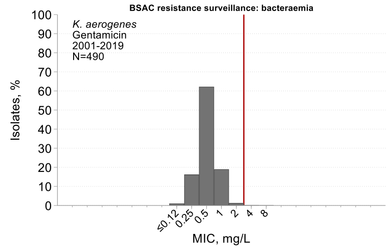

Imipenem

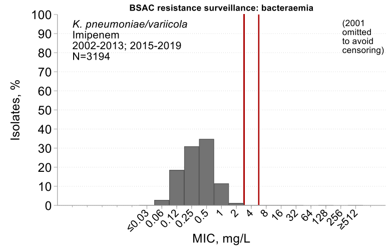

Imipenem

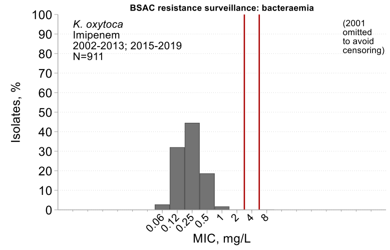

Imipenem

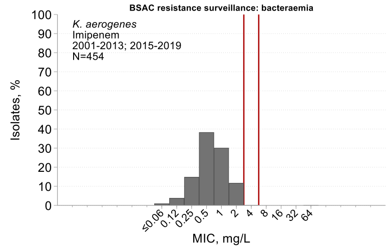

Imipenem/relebactam

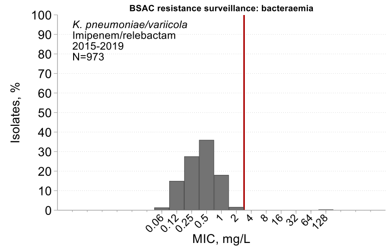

Imipenem/relebactam

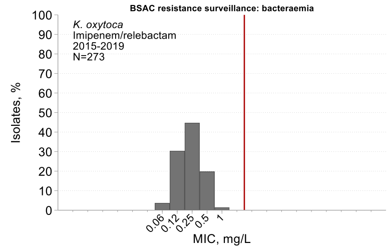

Imipenem/relebactam

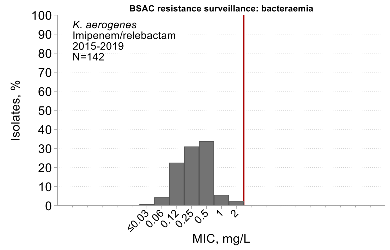

Meropenem

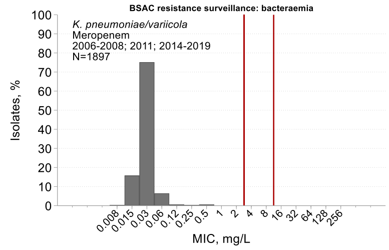

Meropenem

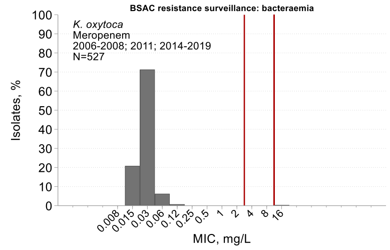

Meropenem

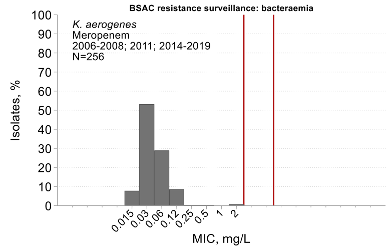

Piperacillin/tazobactam

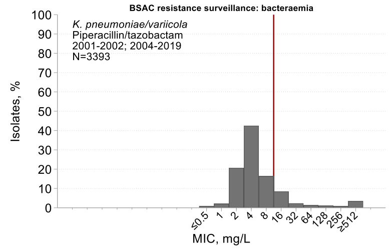

Piperacillin/tazobactam

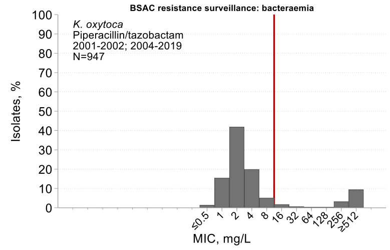

Piperacillin/tazobactam

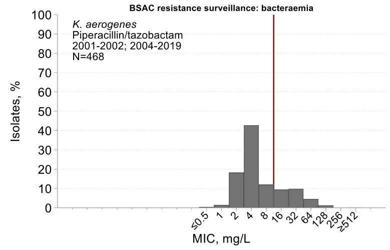

Klebsiella

K. pneumoniae/variicola

Tigecycline

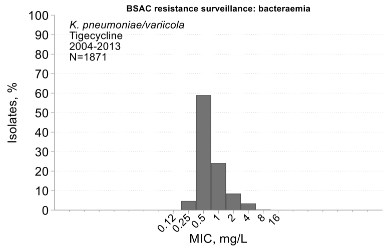

Tobramycin

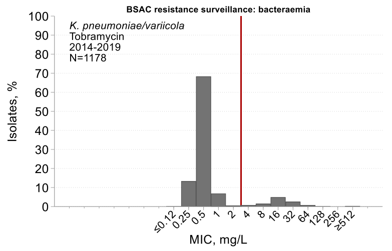

K. oxytoca

Tigecycline

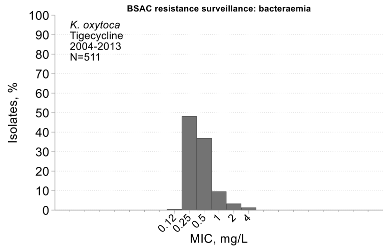

Tobramycin

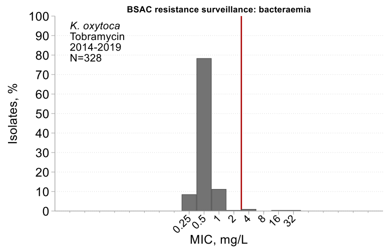

K. aerogenes

Tigecycline

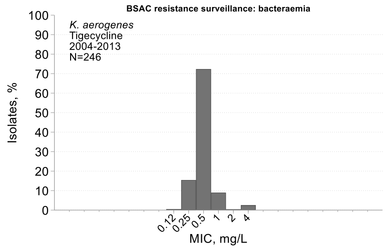

Tobramycin

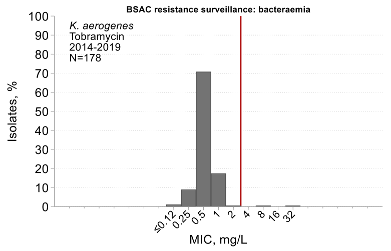

E. cloacae complex

E. cloacae complex

Amikacin

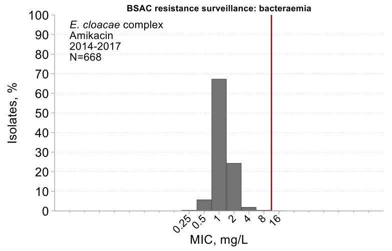

Cefotaxime

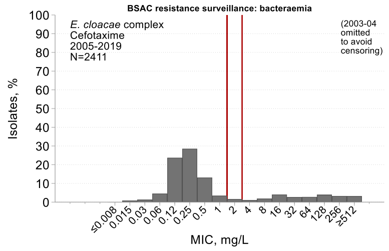

Ceftaroline

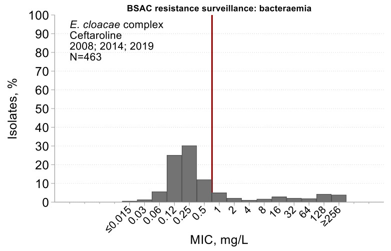

Ceftazidime

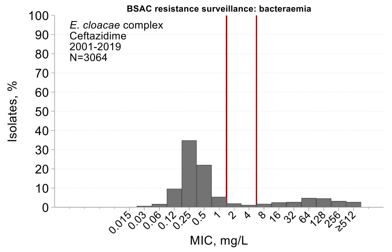

Ceftazidime/avibactam

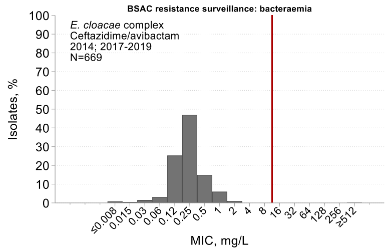

Ceftobiprole

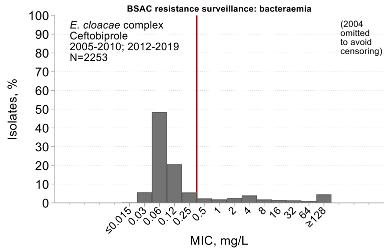

Ceftolozane/tazobactam

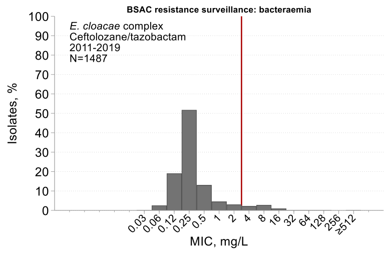

Ciprofloxacin

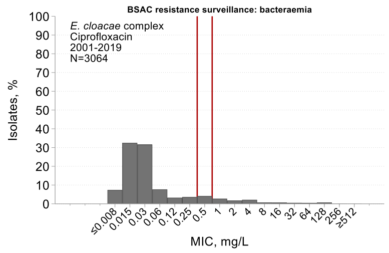

Colistin

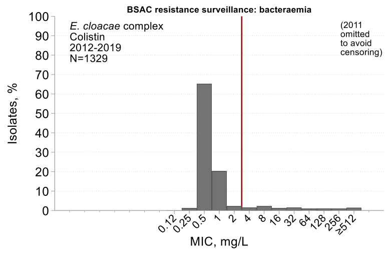

E. cloacae complex

Ertapenem

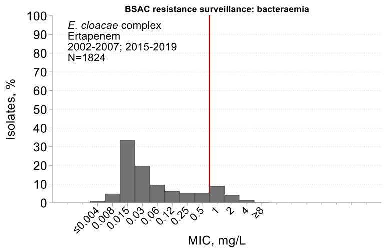

Gentamicin

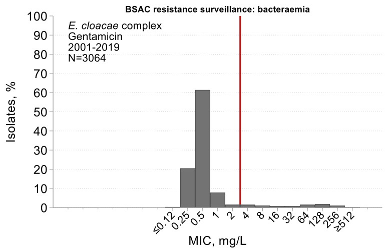

Imipenem

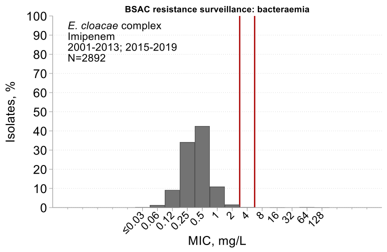

Imipenem/relebactam

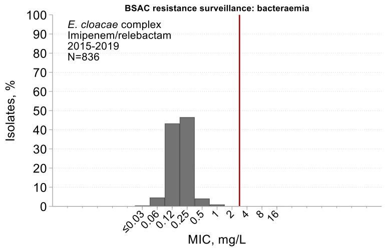

Meropenem

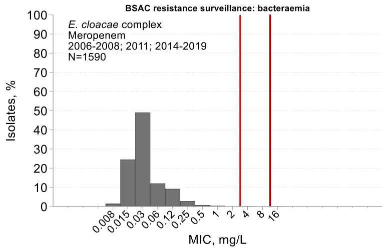

Piperacillin/tazobactam

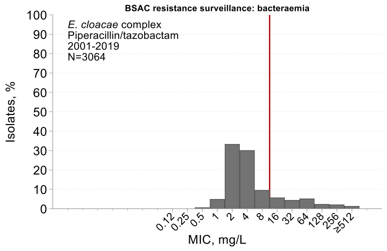

Tigecycline

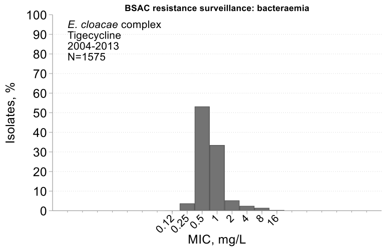

Tobramycin

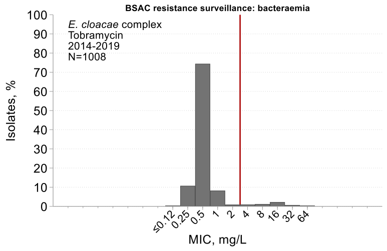

P. mirabilis

Proteus mirabilis

Amikacin

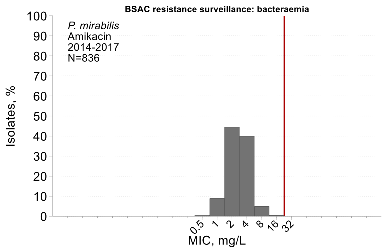

Amoxicillin

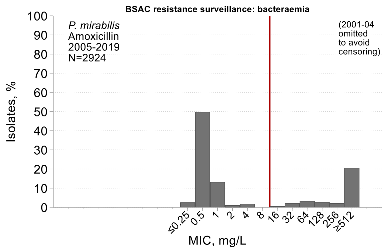

Co-amoxiclav (2:1ratio)

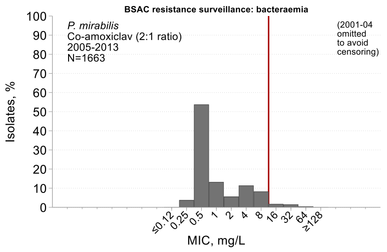

Co-amoxiclav (2mg/Lclavulanate)

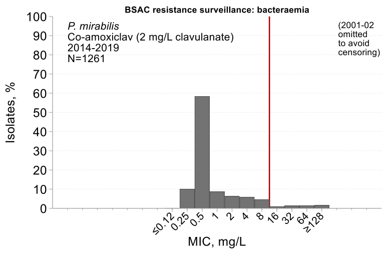

Cefotaxime

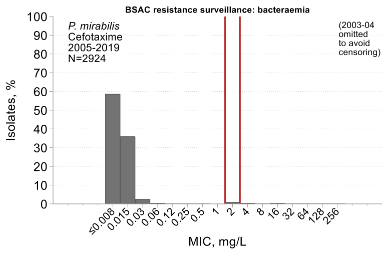

Ceftaroline

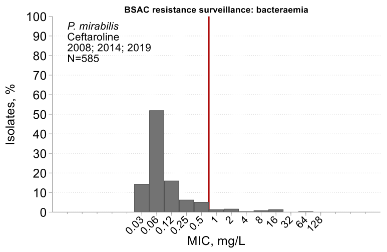

Proteus mirabilis

Ceftazidime

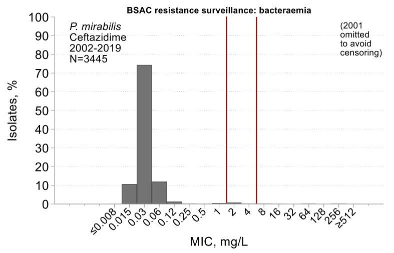

Ceftazidime/avibactam

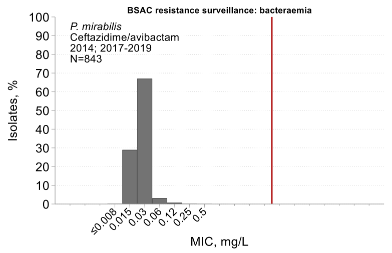

Ceftobiprole

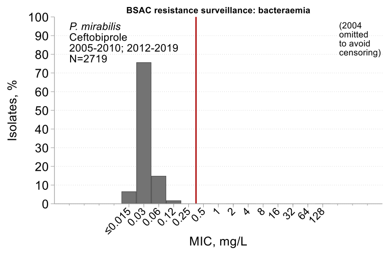

Ceftolozane/tazobactam

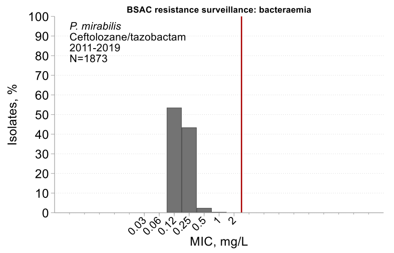

Cefuroxime

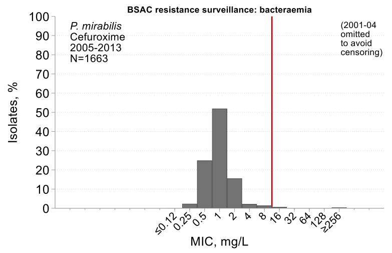

Ciprofloxacin

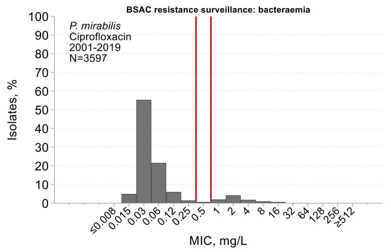

Ertapenem

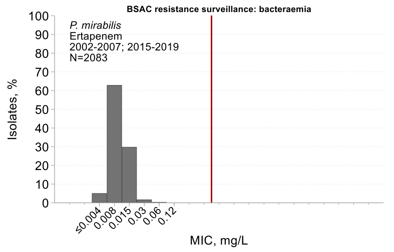

Gentamicin

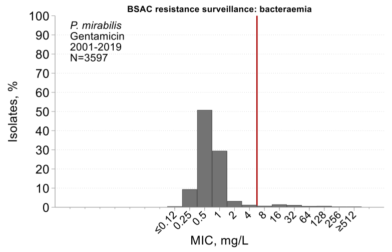

Imipenem

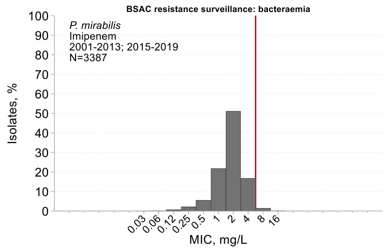

Imipenem/relebactam

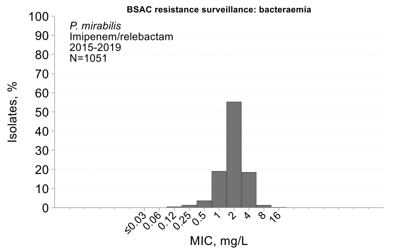

Meropenem

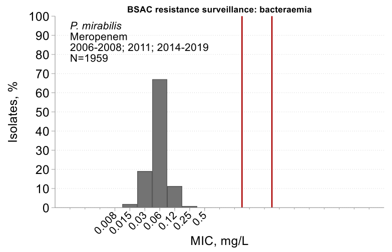

Piperacillin/tazobactam

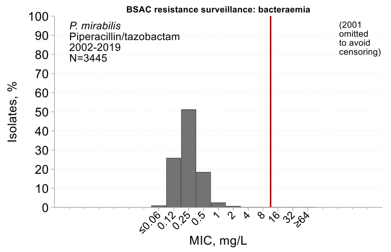

Tigecycline

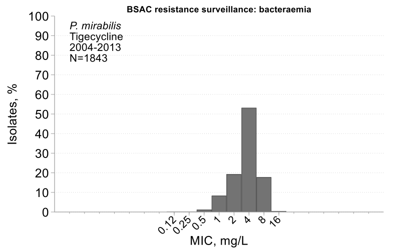

Tobramycin

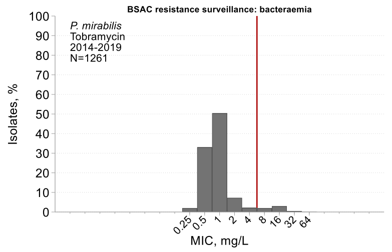

M. morganii

Note small numbers of isolates (<50) in some cases.

Morganella morganii

Amikacin

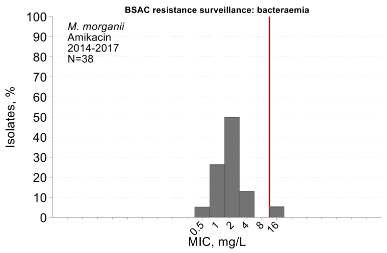

Cefotaxime

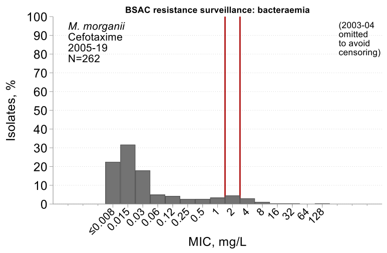

Ceftaroline

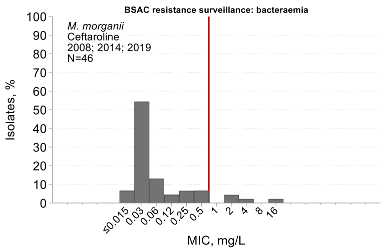

Ceftazidime

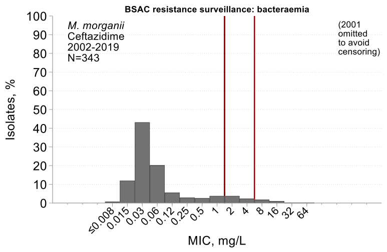

Ceftazidime/avibactam

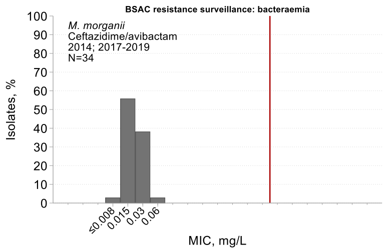

Ceftobiprole

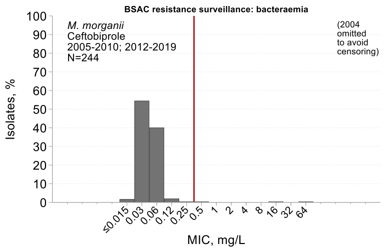

Ceftolozane/tazobactam

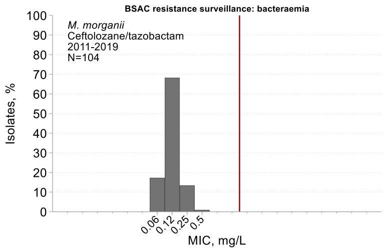

Ciprofloxacin

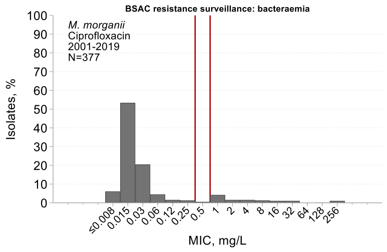

Ertapenem

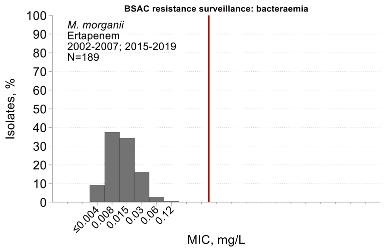

Gentamicin

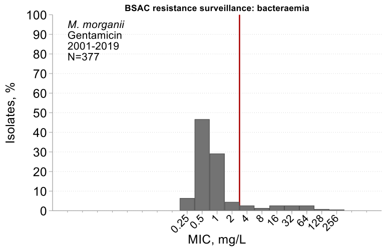

Imipenem

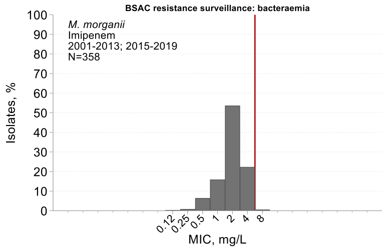

Imipenem/relebactam

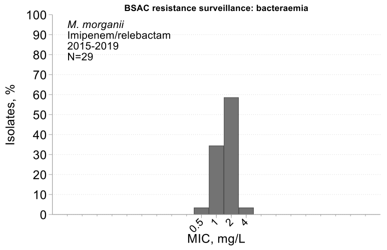

Meropenem

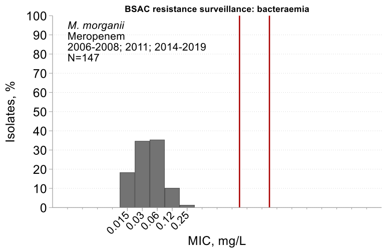

Piperacillin/tazobactam

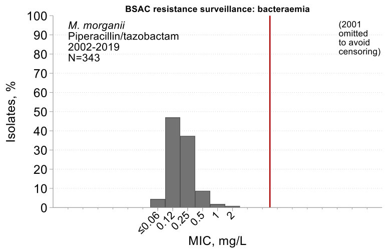

Tigecycline

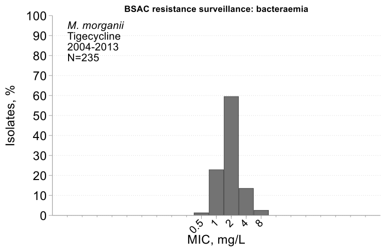

Tobramycin

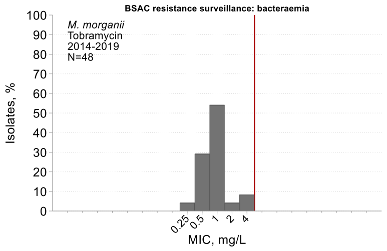

Serratia

S. marcescens	*S. liquefaciens*

Amikacin

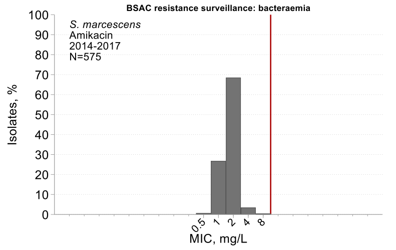

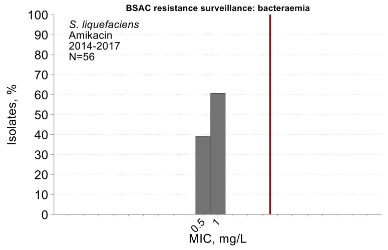

Cefotaxime

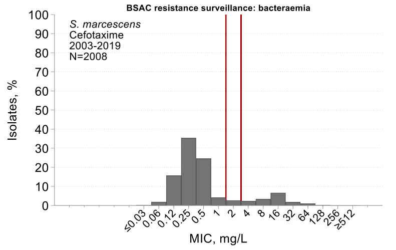

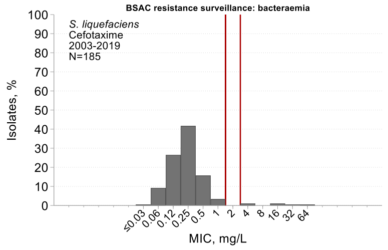

Ceftaroline

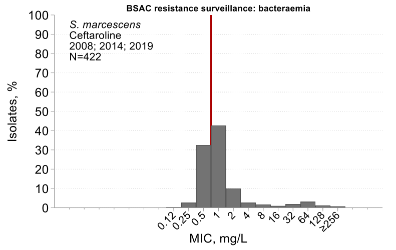

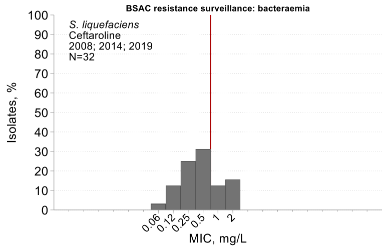

Ceftazidime

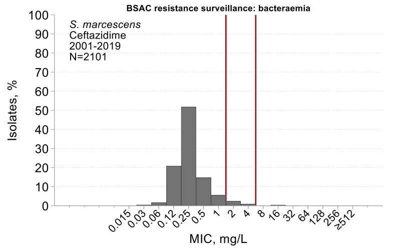

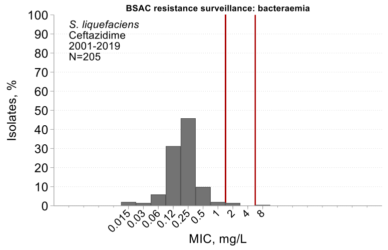

Ceftazidime/avibactam

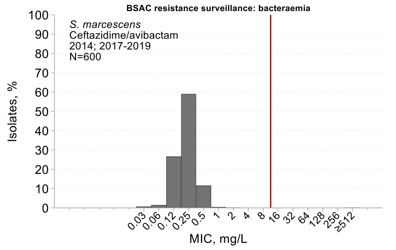

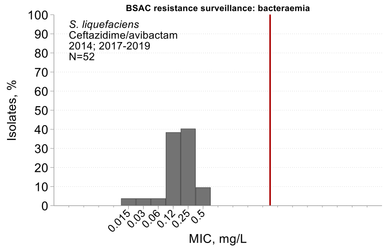

Ceftobiprole

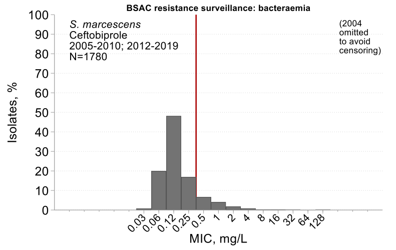

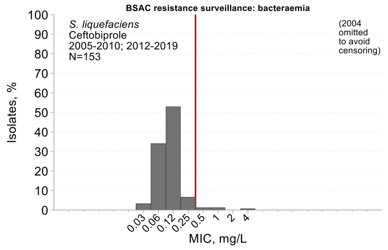

S. marcescens	*S. liquefaciens*

Ceftolozane/tazobactam

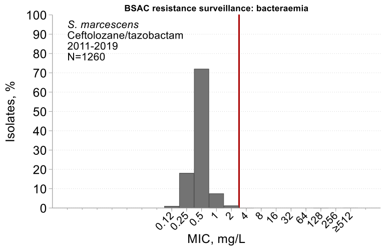

Ceftolozane/tazobactam

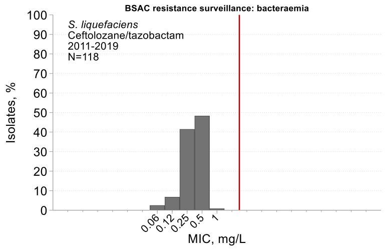

Ciprofloxacin

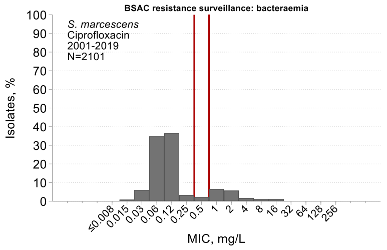

Ciprofloxacin

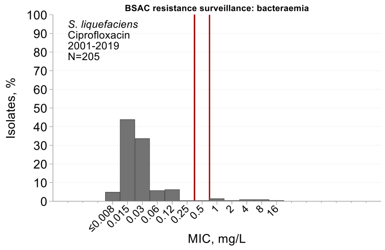

Ertapenem

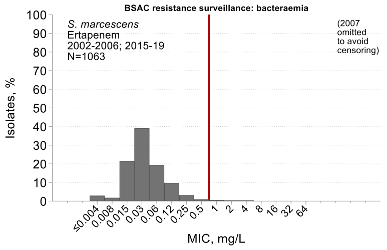

Ertapenem

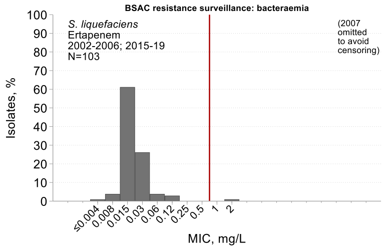

Gentamicin

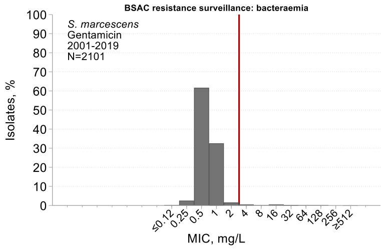

Gentamicin

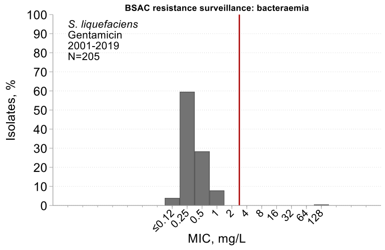

Imipenem

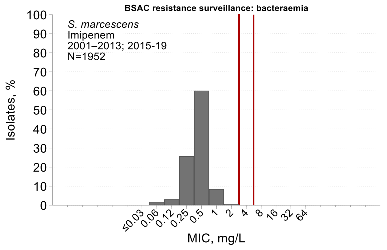

Imipenem

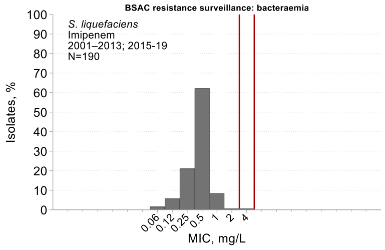

Imipenem/relebactam

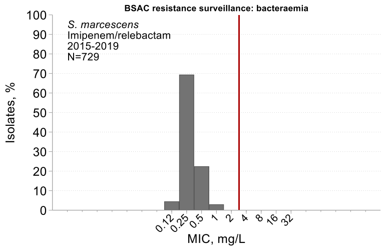

Imipenem/relebactam

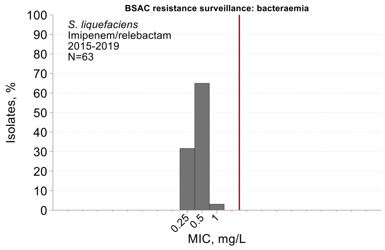

S. marcescens	***S. liquefaciens***

Meropenem

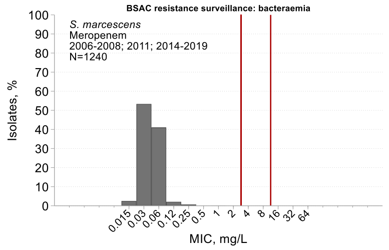

Meropenem

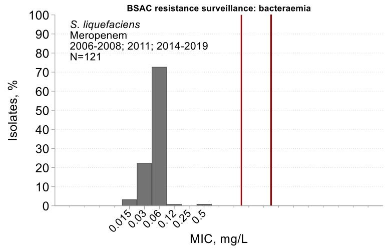

Piperacillin/tazobactam

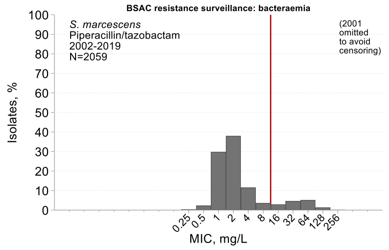

Piperacillin/tazobactam

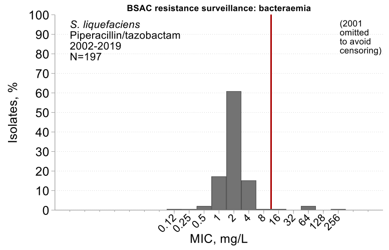

Tigecycline

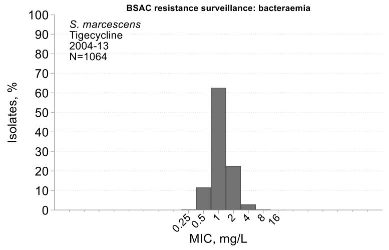

Tigecycline

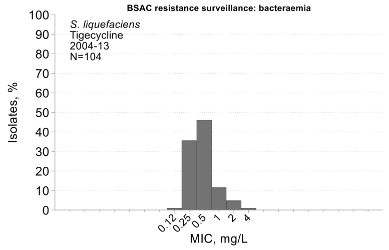

Tobramycin

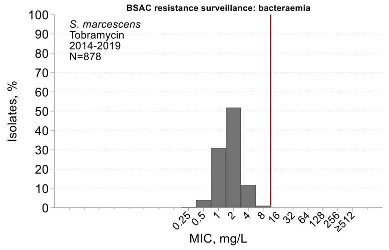

Tobramycin

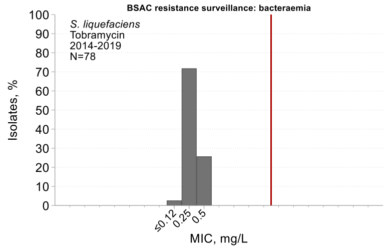

P. aeruginosa

Pseudomonas aeruginosa

Amikacin

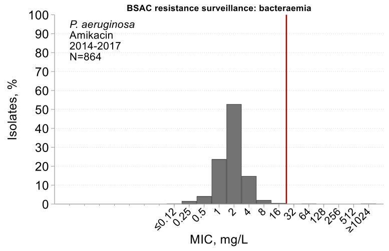

Ceftazidime

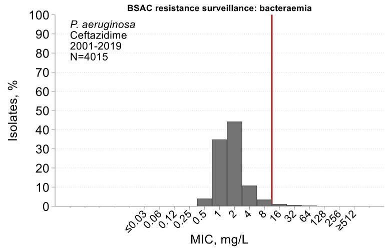

Ceftazidime/avibactam

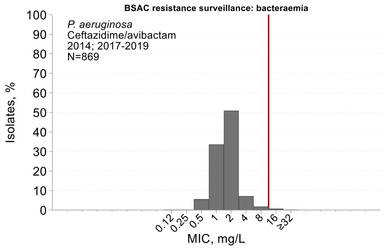

Ceftobiprole

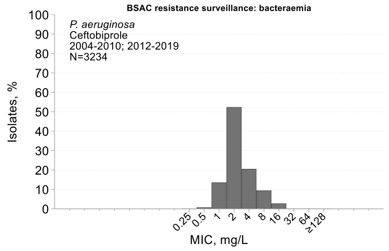

Ceftolozane/tazobactam

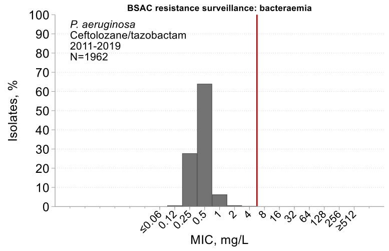

Ciprofloxacin

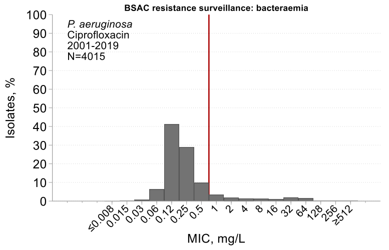

Colistin

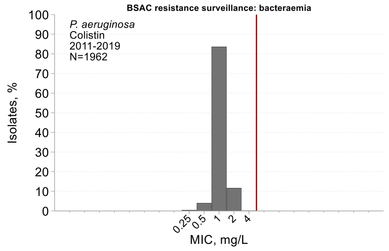

Gentamicin

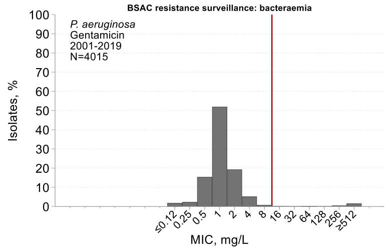

Imipenem

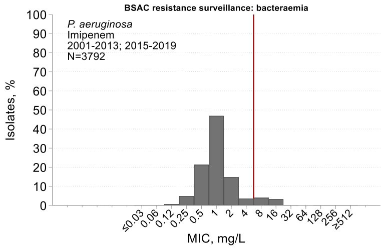

Imipenem/relebactam

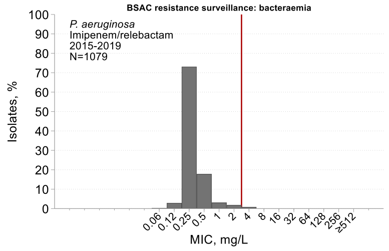

Meropenem

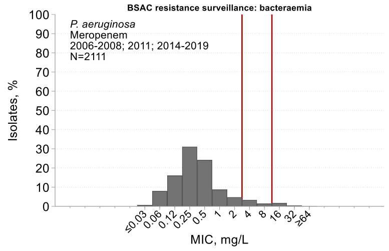

Piperacillin/tazobactam

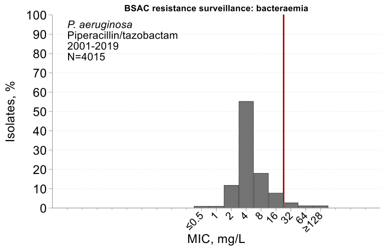

Tobramycin

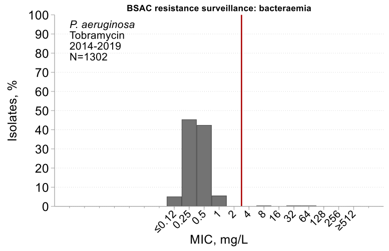

Supplement: dkaf250_Supplementary_Data [file dkaf250_supplementary_data.pdf]
